# Integrative Genomic and Transcriptomic Analysis Reveals Targetable Vulnerabilities in Angioimmunoblastic T‐Cell Lymphoma

**DOI:** 10.1002/ajh.27736

**Published:** 2025-06-13

**Authors:** Alyssa Bouska, Weiwei Zhang, Sunandini Sharma, Harald Holte, Rauf A. Shah, Waseem G. Lone, Mahfuza Afroz Soma, Ruimeng Yang, Xuxiang Liu, Syed Mehmood, Ravneet Singh Chawla, Luca Vincenzo Cappelli, Danilo Fiore, Qiang Gong, Tayla B. Heavican‐Foral, Jeffrey J. Cannatella, Catalina Amador, Aiza Arif, Lynette M. Smith, Soon Thye Lim, Choon Kiat Ong, Andrew L. Feldman, Ming‐Qing Du, Anamarija M. Perry, Laurence de Leval, Timothy C. Greiner, Kai Fu, Gunhild Trøen, Daniel Vodák, Sigve Nakken, Jan Delabie, David Weinstock, Stefano Pileri, Antonella Laginestra, KyeongJin Kim, Utpal Pajvani, Julie M. Vose, Dennis D. Weisenburger, Steven M. Horwitz, Sandeep Dave, Joseph Khoury, Giorgio Inghirami, Wing C. Chan, Javeed Iqbal

**Affiliations:** ^1^ Department of Pathology, Microbiology, and Immunology University of Nebraska Medical Center Omaha Nebraska USA; ^2^ Department of Oncology Oslo University Hospital Oslo Norway; ^3^ KG Jebsen Centre for B‐Cell Malignancies Oslo Norway; ^4^ Department of Pathology, City of Hope National Medical Center Duarte California USA; ^5^ Department of Pathology and Laboratory Medicine Weil Cornell Medical College New York New York USA; ^6^ Department of Molecular Medicine and Medical Biotechnology University of Naples Federico II Naples Italy; ^7^ Department of Pathology and Laboratory Medicine University of Miami Miller School of Medicine Miami Florida USA; ^8^ Department of Biostatistics University of Nebraska Medical Center Omaha Nebraska USA; ^9^ Division of Medical Oncology, National Cancer Centre Singapore/Duke‐NUS Medical School Singapore Singapore; ^10^ Department of Laboratory Medicine and Pathology Mayo Clinic College of Medicine Rochester Minnesota USA; ^11^ Department of Pathology University of Cambridge Cambridge UK; ^12^ Department of Pathology University of Michigan Ann Arbor Michigan USA; ^13^ Institute of Pathology, Department of Laboratory Medicine and Pathology Lausanne University Hospital (CHUV) and University of Lausanne Lausanne Switzerland; ^14^ Department of Pathology and Laboratory Medicine, Roswell Park Comprehensive Cancer Center Buffalo New York USA; ^15^ Department of Pathology Oslo University Hospital Oslo Norway; ^16^ Department of Tumor Biology, Institute for Cancer Research, The Norwegian Radium Hospital University of Oslo Oslo Norway; ^17^ Center for Cancer Cell Reprogramming, Institute of Clinical Medicine University of Oslo Oslo Norway; ^18^ Center for Bioinformatics, Department of Informatics University of Oslo Oslo Norway; ^19^ Toronto General Hospital: University Health Network (UHN) University of Toronto Toronto Ontario Canada; ^20^ Department of Medical Oncology Dana Farber Cancer Institute Boston Massachusetts USA; ^21^ European Institute of Oncology IRCCS Milan Italy; ^22^ Bologna University School of Medicine Bologna Italy; ^23^ Department of Biomedical Sciences, College of Medicine Inha University Incheon Republic of Korea; ^24^ Program in Biomedical Science & Engineering Inha University Incheon Republic of Korea; ^25^ Research Center for Controlling Intercellular Communication (RCIC), College of Medicine Inha University Incheon Republic of Korea; ^26^ Department of Medicine Columbia University New York New York USA; ^27^ Department of Hematology and Oncology University of Nebraska Medical Center Omaha Nebraska USA; ^28^ Lymphoma Service, Department of Medicine, Memorial Sloan Kettering Cancer Center New York New York USA; ^29^ Department of Medicine Duke University Durham North Carolina USA

**Keywords:** cancer genetics, genomics and transcriptomics, lymphomaangioimmunoblastic T‐cell lymphoma

## Abstract

Nodal follicular helper T‐cell (**T**_FH_) lymphoma of the angioimmunoblastic (AITL) subtype has a dismal prognosis. Using whole‐exome sequencing (*n* = 124), transcriptomic (*n* = 78), and methylation (*n* = 40) analysis, we identified recurrent mutations in known epigenetic drivers (*TET2, DNMT3A, IDH2* ^*R172*^) and novel ones (*TET3, KMT2D*). *TET2, IDH2* ^*R172*^, *DNMT3A* co‐mutated AITLs had poor prognosis (*p* < 0.0001). Genes regulating T‐cell receptor (TCR) signaling (*CD28, PLCG1, VAV1*, *FYN*) or activation (*RHOA* ^*G17V*^) or regulators of the PI3K‐pathway (PIK(3)C members, *PTEN, PHLPP1, PHLPP2*) were mutated. *CD28* mutation/fusion was associated with poor prognosis (*p* = 0.02). WES of purified, neoplastic T‐cell (CD3^+^PD1^+^) demonstrated high concordance with whole tumor biopsies and validated the presence of *TET2* and *DNMT3A* in tumor and non‐lymphoid cells, but other mutations (*CD28*, *RHOA* ^*G17V*^, *IDH2* ^*R172*^, *PLCG1*) in neoplastic cells. Integrated DNA‐methylation and mRNA expression analysis revealed epigenetic alterations in genes regulating TCR, cytokines, PI3K‐signaling, and apoptosis. RNA‐seq analysis identified fusion transcripts regulating TCR‐activation (8%), revealed a restricted TCR‐repertoire (α = 87%, β = 72%), and showed the presence of Epstein–Barr virus transcriptome (73%). GEP demonstrated the association of B‐cells or dendritic cells in the tumor milieu with prognosis (*p* < 0.01). RNA‐seq and WES analysis of 12 AITL‐patient‐derived‐xenografts (PDX) showed that bi‐allelic *TET2* and *DNMT3A* mutations or sub‐clonal mutations (*PLCG1, PHLPP2*) propagated in sequential passages, and gene signatures related to **T**_FH_ and **T**_CM_ (central‐memory) were well‐maintained through passages. Gene expression signatures associated with late PDX passages (3rd–5th) were enriched with proliferation and metabolic reprogramming‐related genes and predicted prognosis in an independent AITL series. Low *PHLPP2* mRNA expression predicted poor prognosis (*p* = 0.05) and engineered *PHLPP2* or *TET2 loss* in CD4^+^ T‐cells showed enhanced PI(3)K activation, thus uncovering a therapeutic target for clinical trials.

## 1 Introduction

Angioimmunoblastic T‐cell lymphoma (AITL) is a distinct clinicopathological entity of peripheral T‐cell lymphoma (PTCL), associated with an aggressive clinical course with a 5‐year progression‐free survival of ≤ 18% [1, 2]. While AITL accounts for ~35% of non‐cutaneous PTCL in North America and Europe, the incidence is increasing in some Asian countries [3, 4, 5]. AITL has a broad spectrum of clinical and pathologic features and usually presents as a systemic nodal disease of post‐thymic T‐cells with prominent proliferation of blood vessels and follicular dendritic cells (FDCs) [6, 7]. AITL is a follicular T‐helper cell derived lymphoma (TFHL) and the current WHO classification also recognizes two other related PTCL entities, follicular helper T‐cell lymphoma (F_H_‐TCL) and nodal PTCL with T_FH_ phenotype (PTCL‐T_FH_), as supported by their shared mutational profile with AITL [7]. The International Consensus Classification (ICC) also recognizes two other T_FH_ lymphomas besides AITL, follicular‐type and TFHL‐NOS [8]. These lymphomas are now grouped with AITL in the 2024 revised World Health Organization (WHO) classification in the new category of nodal T‐cell lymphoma of T_FH_‐cell origin [9]. The neoplastic cells are typically medium‐sized with clear cytoplasm, tend to form small clusters around high endothelial venules, and express several T_FH_ cell markers such as programmed death‐1 (PD‐1), Inducible T‐cell co‐stimulator (ICOS), cytoplasmic SLAM‐associated protein (SAP), BCL6, and c‐MAF, or proteins associated with T_FH_ biology (e.g., CD10, CXCL13, CXCR5, CD154/CD40L). AITL is often preceded by autoimmune manifestations and has a remarkably immunosuppressive tumor microenvironment (TME) with frequent Epstein–Barr virus (EBV) infected large B‐cell blasts [10], sometimes mimicking Reed‐Sternberg cells, which can make correct diagnosis more challenging [11]. In addition to the clonal TCR gene rearrangement, a clonal immunoglobulin (IG) gene rearrangement is observed in 10%–20% of AITL.

While the accurate diagnosis of AITL requires an integrated approach, incorporating clinical, morphologic, immunophenotypic, and molecular findings, an improved understanding of the genome may not only aid in accurate diagnosis, but also reveal novel therapeutics, and is a step towards the unmet need of precision medicine in AITL. We have used gene expression profiling (GEP), miRNA profiling [12], high‐resolution genomic DNA copy number aberrations (g‐CNA) analysis, and next‐generation sequencing (NGS) to elucidate AITL pathobiology [13, 14, 15, 16, 17]. These studies resulted in a robust diagnostic molecular classifier and a prognostic gene‐signature that mainly reflects the tumor microenvironment [13, 18]. We and others have also identified frequent mutations in genes regulating the epigenome (*TET2, DNMT3A*, *IDH2* ^*R172*^) [14, 16, 17], or TCR signaling (*CD28, PLCγ1*, *VAV1*) or T‐cell activation (RhoA^G17V^) [16, 19, 20, 21]. We also observed recurrent g‐CN gain/amplification of chromosome‐5 and ‐21 associated with IDH2^R172^ mutations, and g‐CN losses targeting PI3K/mTOR pathways [22]. However, to date, comprehensive genetic and transcriptomic characterization of AITL is established from limited cases not exceeding 25 AITL [23, 24, 25, 26]; herein, we assembled a large AITL cohort through a consortium of hospitals in Asia, Europe, and North America, and performed whole‐exome sequencing (WES), transcriptional and methylation profiles and generated AITL patient‐derived xenografts (PDX) and elucidated novel targetable mechanisms using engineered CD4^+^ T‐cells.

## 2 Material and Methods

See complete details in the Supporting Information methods section.

### 2.1 Patient Specimen

174 AITLs with WES, RNA‐seq, and/or methylation profiling and diagnosed by standard morphological and molecular techniques according to the current WHO classification [7] are described in Tables S1, S2 and Figure S1A (see Supporting Information methods). This study was approved by the institutional review boards of contributing sites.

### 2.2 DNA and RNA Isolation and Library Preparation for High‐Throughput WES and RNA‐Seq Analysis

Total RNA and g‐DNA were extracted using the DNeasy Blood and Tissue Kit (Qiagen Inc., MD) and quality and quantity were assessed using the Agilent Bio‐analyzer and Qubit fluorometric quantitation. The library preparation, sequencing, and analysis were done as previously described [22, 27, 28] (see Supporting Information methods).

### 2.3 Genome‐Wide Methylation Profiling

Genome‐wide methylation profiling was performed using the Infinium HumanMethylation450 BeadChip array (Illumina, San Diego, CA) according to the manufacturer's protocol and integrated with additional AITL cases from our earlier series [29](See Supporting Information methods).

### 2.4 Genomic Characterization of AITL Patient‐Derived Xenografts

This section is described in the Supporting Information methods.

### 2.5 In Vitro Functional Analysis of *PHLPP2* and *TET2* ‐Loss

The experimental design is described in the Supporting Information methods.

### 2.6 Overall Survival Analysis

Statistical analysis is described in the Supporting Information methods.

### 2.7 Data Sharing

Data will be deposited in the GDC database or available by contacting the authors.

## 3 Results

### 3.1 Clinical Characteristics

The basic clinicopathological characteristics of the cases are shown in Tables S1, S2, and a representative case is shown in Figure S1B. Of the AITL cohort analyzed for genetic or transcriptomic analysis (*n* = 109 of 174 with clinical outcome information), 62% were males and 38% females, with an average age of 63 years (range: 19–91 years, median = 64.7, 18% below age 50). The median follow‐up time was 22.8 months (0.07–264 months) and median follow‐up time for live patients was 45.3 months. This retrospective cohort of AITL was mostly treated with polychemotherapy, and 90% (67/75) of the cases with therapy information were treated with curative intent. The 5‐year OS was 36.4%, consistent with previously reported clinical experience in AITL patients [1, 13, 30, 31]; however, AITL patients < 50 years old (*n* = 20) were significantly associated with good OS (*p* = 0.02), though in cases < 60 years old the outcome difference was not significant (*p* = 0.22), and females had comparatively better OS than males (*p* = 0.07) (Figure S1C–E).

### 3.2 Identification of Somatic Mutations and Associated Oncogenic Pathways

WES was performed on 36 AITLs with paired germline tissue and 18 additional paired AITLs (tumor–germline) from earlier studies [19, 26, 32, 33] were combined with WES of 65 AITLs lacking germline tissue (unpaired) to improve mutation discovery (Figure S2). Low sequencing‐depth cases were excluded for mutation frequency estimations; thus, 90 cases were used for frequency calculations (48 paired, 42 unpaired details in Supporting Information results and Table S3). Validation of recurrent mutations in a subset of AITLs (*n* = 14) using corresponding targeted‐amplicon sequencing [17, 29], and RNA‐seq data (*n* = 31) revealed high concordance with WES data (81.5%, Table S4) and indicated high confidence in SNV detection.

**FIGURE 1:**
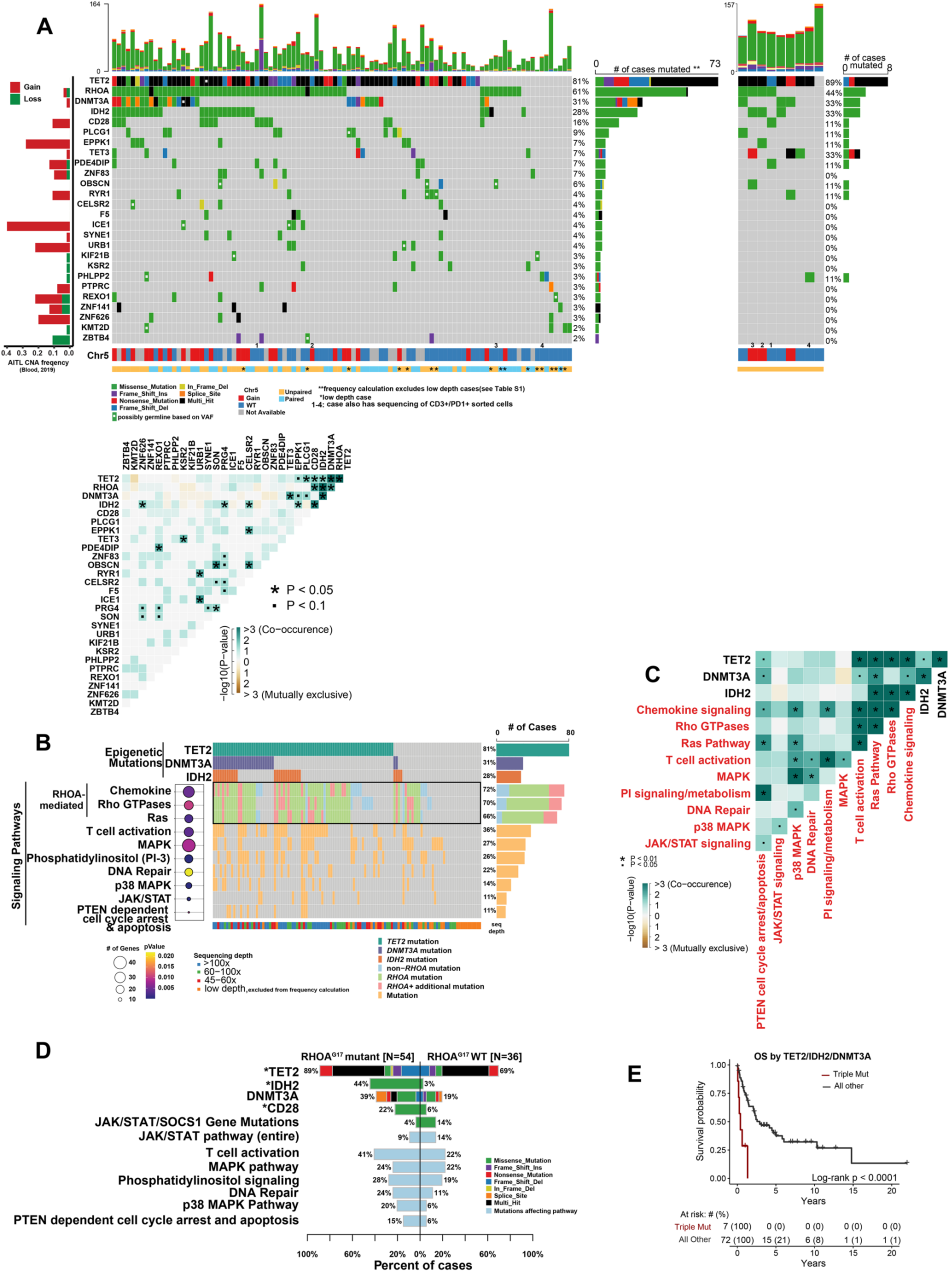
Mutations analysis in AITLs. (A) The oncoplot illustrates recurrent mutations identified through WES. The genes were selected by assessing their expression levels in normal T‐cells via RNA‐seq data, considering their functional relevance. Below, the chromosome‐5 copy number (CN) status, uniquely associated with AITLs, is presented and whether germline was also sequenced. The left oncoplot represents the 100 cases (of 119 sequenced) that had mutations in the depicted genes. The right oncoplot includes nine cases that were sorted for CD3+/PD1+ cells to enrich the tumor fraction. Four cases with WES sequencing on both the whole section and enriched tumor cells are noted. Lower panel: Plot of mutations that either co‐occur or are mutually exclusive using pairwise Fisher's exact tests for significance (*p*‐value). (B) Association of mutant genes in relation to the signaling transduction pathways. The mutated gene was placed within biological pathways using David.V6.7. A waterfall plot of the top three epigenetic mutations and significantly affected pathways are shown. On the left, the bubble plot illustrates the enrichment significance (*p*‐value) and number of genes within affected pathways. The three signaling pathways (i.e., Chemokine, RhoGTPases, and Ras in black box) were dominated by *RHOA* ^*G17V*^ (green color), however mutations in other genes in the pathways are colored blue. (C) Pairwise Fisher's exact tests were used to reveal correlation between epigenetic dysregulation (i.e., genes in black text) and other signaling pathways (red text) that demonstrate either co‐occurrence or mutual exclusivity. (D) Bar plot illustration of frequency of mutations in the genes/pathways in AITLs with *RHOA* ^*G17V*^ versus WT (Wild Type) cases. The asterisk indicates mutation with a significant difference between two groups (Fisher's exact test, *p* < 0.05). (E) Association of OS in AITLs carrying triple mutation (*TET2, IDH2* ^*R172*^, *DNMT3A*) versus WT. Significantly inferior is associated with cases having triple mutations. [Color figure can be viewed at wileyonlinelibrary.com]

**FIGURE 2:**
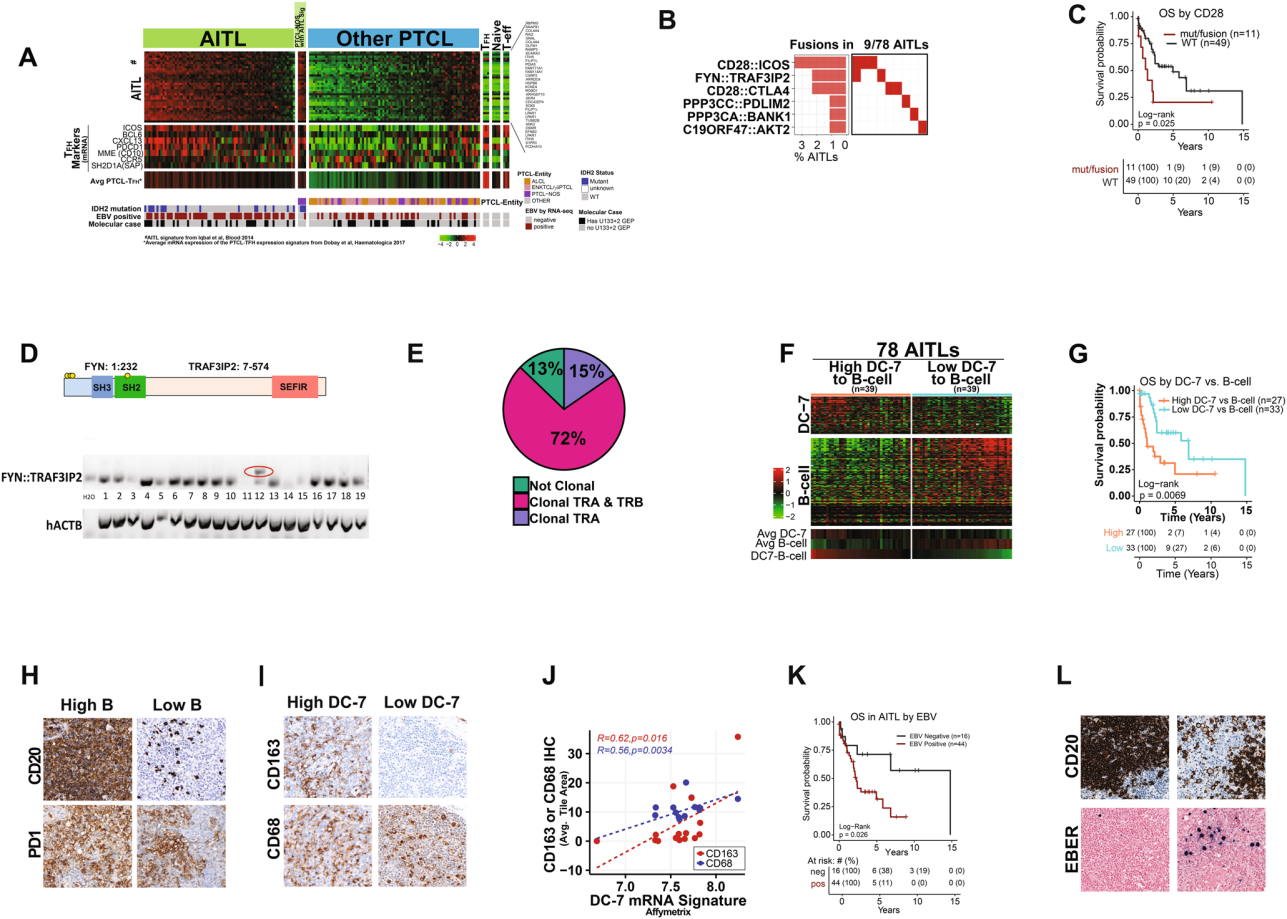
Transcriptomic and tumor microenvironment analysis in AITL. (A) Evaluation of pre‐defined AITL mRNA diagnostic signature (Iqbal et al., Blood 2014) utilizing RNA‐seq data of AITL versus other PTCLs (i.e., PTCL‐NOS, ALCL, ENKTCL, and γδ‐PTCL). The diagnostic signature is also assessed in normal **T**_FH_, naïve CD4+ T‐cells, and T‐effector cells. EBV transcript was identified from RNA‐seq data. Four PTCL‐NOS (now classified as n‐TFHLs) showed significant association with the AITL diagnostic signature and 3 of 4 showed IDH2^R172^ variant. These cases also have high expression of T_FH_ specific genes, as noted in the heatmap. The lower section of the figure includes information on IDH2 mutations, EBV status, and the corresponding GEP data using HG‐U133plus2 platform. (B) Identification of in‐frame fusions in transcripts involved in the TCR signaling using RNA‐seq data utilizing fusion‐catcher. These fusions were also evaluated in normal T‐cells and not found. (C) Association *CD28* genetic alterations (mutation/fusion) with OS demonstrate inferior OS associated with *CD28* alterations. (D) Schematic of the *FYN:TRAF3IP2* fusion identified by RNA‐seq and real‐time qPCR product of *FYN‐TRAF3IP2* fusion (upper) or control primers (beta‐actin) in AITL cases. Fusion was verified by Sanger sequencing (see Figure S5E). (E) Assessment of T‐cell clonality using RNA‐seq data showed TCR‐α and TCR‐β clonal fraction in 87% of cases as indicated by Pie Chart. (F) Heatmap evaluating the dendritic‐cell mRNA signature (designated as (DC‐7, https://lymphochip.nih.gov/signaturedb/) and the B‐cell Expression Signature (https://lymphochip.nih.gov/signaturedb/, Newman A.M. et al., Nat. Methods 2015) in 78 AITLs. Mean expression levels of both signatures and the difference between the two are shown below the main heatmap. (G) Association of OS with the DC‐7 signature expression vs. B‐cell signature expression. The 78 AITL cases with RNA‐seq were divided into two halves (High DC‐7 vs. B‐cell signature and Low DC‐7 vs. B‐cell Signature) and OS assessed in the 60 cases with outcome data. AITL with higher DC‐7 vs. B‐cell mRNA signature are associated with poor prognosis (*p* = 0.0069). (H) Immunostains (×40) of CD20 and PD1 in a representative AITL case with high B‐cell content based on CIBERSORT (left) and low B‐cell content based on CIBERSORT (right). (I) Immunostains (×40) of myeloid markers CD68 and CD163 in a representative high DC‐7 and low DC‐7 case. (J) Association of DC‐7 mRNA signature with two representative IHC biomarkers (CD68 or CD163) quantified by QuPath software. A significant association with (CD163: *R* = 0.62, *p* = 0.016; CD68: *R* = 0.56, *p* = 0.0034) was observed. Correlation coefficients were calculated by spearman correlation and the dashed line represents the linear regression trendline. (K) Association of OS with EBV status assessed using RNA‐seq data. Significant association of EBV transcript with poor prognosis (*p* = 0.026) was noted in these cases. (L) CD20 IHC and EBER in situ hybridization (ISH) for one representative EBER‐positive (right) and one representative EBER‐negative (left) case. [Color figure can be viewed at wileyonlinelibrary.com]

The most frequent variants included *TET2* (81%), *RHOA* ^*G17V*^ (61%), *DNMT3A* (31%), *IDH2* ^*R172*^ (28%), *CD28* (16%), and *PLC*γ1 (9%), consistent with our and other earlier studies [16, 17, 34], and newly defined mutations were either tumor suppressor genes or chromatin modifier genes (Figure 1A). To summarize functional categories of the mutant genes, we used in silico gene ontology (GO) and pathway enrichment analysis (DAVID, v6.7), and the major oncogenic perturbations targeted epigenetic reprogramming, TCR activation, PI(3)K and RAS‐MAPK–ERK signaling (Figure 1B, Table S5). While genes regulating DNA methylation, including novel *TET3* mutations, were the most frequent ones, genes involved in histone modification (*EP400*, *SETD2*, and *KMT2D*) were infrequent, suggesting that altered DNA methylation, rather than chromatin reorganization, is a predominant oncogenic driver in AITLs. Interestingly, *KMT2D* mutations were relatively higher in AITLs lacking *TET2* mutations (7.7%; (3/39) vs. 1.3%; (1/80), Fisher's exact test, *p* = 0.1). Epigenetic dysregulation was followed by mutations affecting TCR signaling, mainly due to mutations of *CD28* and *PLCγ1*, which rarely co‐occurred, and infrequent mutations of anchor/adaptor proteins (*VAV1/3*), the SRC kinase *FYN*, novel genes associated with TCR activation (e.g., *PTPRC/CD45* [35], *AKAP9* [36]) or downstream of TCR signaling including regulators of intracellular calcium (Ca^2+^) signaling. Several key regulators of the PI3K pathway, including lipid phosphatases (*PTEN, PHLPP1, PHLPP2*), catalytic (*PIK3CA*/P110α, *PIK3CD*/P110δ, *PIK3C2α*, *PIK3C2β*, *PIK3C2*γ) and regulatory (PIK3R5/P101, PIK3R3/p55γ) subunits, or adenyl cyclase pathway members (*INPPL1*(*SHIP2*), *INPP4A, INPP5J, INPP5K*) were mutated in 26% of AITLs (Figure 1B,C). Mutations affecting the p38/MAPK pathway (*MEF2A, MAP2K3, MAP3K1, MAP3K4, MAP3K6, MAP3K9, MAP4K3, MAPK1, MAPK7*, and *MAPKAPK2*) and JAK–STAT pathway (e.g., JAK2/3 *STAT1/3/5B*) were detected in 14% and ~ 10% AITLs, respectively. Interestingly, other than *RHOA* ^G17V^, infrequent mutations in several Rho‐GEFs regulators (e.g., *BCR, ARHGEF1/2/10L/11/17/18/25/28/38/40*), *ARHGEF30*(*OBSCN*), or RAS‐regulators (*KSR2, RAC1, GRB2, SOS1 SHC1, TIAM1, TIAM2*) occurred in > 20% of AITLs. While significant co‐occurrence of epigenetic regulators with *RHOA* ^*G17*^ is known [19, 32, 33], *RHOA* ^*G17V*^ also showed significant association with *CD28* mutations [37], but not with *PLCγ1*, suggesting different cooperative interaction in aberrant TCR signaling (Figure 1A, right‐panel). AITLs lacking *RHOA* ^*G17V*^ showed mutations of genes regulating proximal JAK/STAT signaling (e.g., *JAK2*, *JAK3*, *STAT1, STAT3, STAT5B, SOCS1*; Figures 1D and S3A). Interestingly, a *STAT3* mutation was observed in a PDX model at passages 1 and 3 (Figure S3A). GEP analysis of the cases with available data identified enrichment JAK–STAT dependent signaling (e.g., *IFN‐γ, IL12*, and *STAT4*; Figure S3B). While *RHOA* mutation showed no association with clinical outcome, *CD28* mutation showed a non‐significant trend with worse OS (*p* = 0.12, Figure S3C,D, see also Figure 2C with *CD28* fusion included). Twelve AITL cases were identified carrying three mutations (*TET2, IDH2* ^*R172*^, *DNMT3A*) and the seven cases with available overall‐survival data showed significantly inferior clinical outcome (Figure 1E).

To validate whether recurrent AITL mutations are tumor‐specific, we performed flow sorting for 10 AITL including neoplastic‐cells (CD3+/PD1+), myeloid (CD68+/CD11B+), and B‐cells (CD3‐/CD79B+) and WES was performed on the tumor enriched cell fraction for nine AITLs (Figure S4A, Table S3). TCRγ rearrangement analysis showed clear clonal peaks in tumor fractions in eight sorted AITLs (Figure S4B). Four AITLs had WES from the whole tissue‐section, which confirmed the presence of the identified mutations and demonstrated an increase in the VAF of the variants in the sorted samples (Figure S4C,D). Moreover, the increased sequencing depth and tumor enrichment identified additional variants, and mutations in *TET2* and/or *IDH2* ^*R172K*^ were detected in three cases that were previously unable to be detected in the whole tumor sample. Overall, mutated genes identified in the tumor‐enriched cohort were concordant, with recurrent variants identified in *TET2*, *DNMT3A*, *RHOA*, *IDH2*, and *TET3* (Figures 1A and S4E). To confirm tumor specificity, we assessed the mutation in the myeloid and B‐cell fraction by Sanger sequencing and found that *IDH2*, *RHOA*, *CD28*, and *PLCγ1* were not detected in the myeloid and B‐cell fraction, but only in tumor fractions (Table S6, Figure S4F). Alternatively, and in accordance with previous studies, *TET2*, *DNMT3A*, and *TET3* were sometimes observed in the myeloid or B‐cell fraction in addition to the tumor indicating it was likely an early founding mutation that occurred in a progenitor cells [38, 39]. In other cases, however, *TET2*, *DNMT3*A, and *TET3* variants, appeared to be tumor specific.

### 3.3 Transcriptomic Analysis and Associations With Genetic Alterations

We performed RNA‐seq analysis (*n* = 74) and assessed a previously GEP‐defined AITL molecular diagnostic signature [40] versus other PTCLs (*n* = 62) [16, 19, 27]. Eighty‐eight percent (21/24) of AITLs previously classified by the HG‐U133plus2 platform (Affymetrix Inc) had similar high expression of the AITL diagnostic signature (Table S7), and 90% (45 of 50) of the cases only profiled by RNA‐seq had high AITL molecular signature expression, while one case was borderline (Figures 2A and S5A). Consistent with previous findings from expression profiling of PTCL‐NOS [40], 4 of the PTCL‐NOS cases expressed the AITL signature, with three cases also having IDH2^R172^ (Figure 2A). High expression of T_FH_‐PTCL signatures, as defined by Dobay et al. [34] or T_FH_ biomarkers [7] were also observed (Figure 2A). These cases were from a published dataset [19] and would now likely be called n‐TFHLs. Thus, the 74 diagnostic AITL cases plus the 4 with the AITL signature were combined for a total of 78 cases used in subsequent analyses. GSEA identified significant enrichment of TCR signaling and other oncogenic pathways (e.g., NF‐κB, MYC), or transcription factor signatures related to T‐cell differentiation (KLF2, FOXO3, FOXOP3, and RUNX1 regulated) in AITLs compared to other PTCLs (Figure S5B,C). Several pathways with mutations including PI3K‐AKT, TCR signaling, and MAP kinase pathway (Figure 1B) were also enriched in AITL by expression analysis.

RNA‐seq data identified fusion transcripts, and several in‐frame hybrid transcripts involving TCR signaling genes were identified, including proximal regulators (e.g., *CD28::ICOS, FYN::TRAF3IP2, CD28::CTLA4*). These fusion transcripts were present in non‐overlapping cases and identified in ~10% of AITLs, but not in normal T‐cells (Figure 2B). *CD28::CTLA4* and *CD28::ICOS* fusions have been reported by us [16, 41]. *CD28* fusion cases show elevated *CD28* expression (Figure S5D, Student's *t*‐test, *p* = 0.11). Though numbers were small, *CD28* fusions and/or mutation cases showed worse outcome (Figure 2C, log‐rank 0.025), which agrees with the previous findings [42]. *FYN::TRAF3IP2* fusion was previously reported to be frequent in AITLs (44%, 4/9 cases) [43]. Unexpectedly, the fusion was infrequent (~3%; 2/78) in the current, larger AITL cohort (Figure 2D). We further examined the cDNA from AITLs not in the RNA‐seq cohort using PCR primers described in the earlier report [43] and confirmed the low frequency (1/19, Figures 2D and S5E).

We identified major TCR clones using the MiXCR algorithm, as estimated by the expression of dominant TCR‐α or ‐β transcripts [27], demonstrating evidence of TCR clonality in 87% of AITLs, consistent with earlier findings [44] (Figures 2E and S5F). Approximately 15% showed only clonal TCR‐α, but not TCR‐β. Assessment using the top two TRA clones versus mutation VAF showed high correlation with *RHOA* ^*G17V*^ and *IDH2* ^*R172*^ indicating the tumor specificity of these mutations, but was skewed with *TET2* and *DNMT3A* mutations, suggesting their presence in progenitor cells in some AITLs (i.e., clonal hematopoiesis of indeterminate potential: CHIP) [38] (Figure S5G).

### 3.4 Association of Tumor Microenvironment (TME) With Prognosis

In silico analysis of TME heterogeneity using CIBERSORT [45] and the Lymphoid Signature Database [46] revealed enrichment of myeloid cells (e.g., dendritic, macrophages) and an inverse correlation between a B‐cell and myeloid‐rich TME (termed DC‐7) (Figures 2F and S5H). Cases with a high DC‐7 to B‐cell signature had a poor outcome (log‐rank test, *p* < 0.01, Figure 2G). In addition, high B‐cell signature mRNA expression was associated with good prognosis (log‐rank test, *p* < 0.01, Figure S5I) and unfavorable clinical outcome with myeloid signatures (log rank test, *p* = 0.033, Figure S5J), as in previous studies [27, 40]. The B‐cell signatures were validated using CD20 IHC and dendritic signatures (DC‐7)‐signature using representative myeloid/macrophage IHC biomarkers (e.g., CD68 or CD163) in a second AITL cohort with HG‐U133plus2 GEP data (Figure 2H,I). Both CD20 and total‐B cells estimated by mRNA signatures and CD68 or CD163 and DC‐7 mRNA signature showed significant correlations (Figure S5K, 2J, *p* = 0.016, *p* = 0.0034).

We analyzed whether B‐cell proliferation in TME is associated with clonal B‐cell expansion using IGH rearrangement analysis and observed ~28% (22/78) AITLs with clonal IGH, consistent with earlier findings [47] (Figure S5L–N). Total IGH counts analyzed by MiXCR correlated significantly with CD20 mRNA expression (Figure S5O). We investigated whether this B‐cell expansion is associated with EBV reactivation [48] and found 70% (57/78) AITLs have EBV transcripts in RNA‐seq (Figure S5P), but showed no association with B‐cell content by CD20 mRNA or IGH counts (Figure S5Q,R), indicating that B‐cell clonal expansion is independent of EBV. EBV transcriptome expression (≥ 10 counts) showed an association with inferior survival (Figure 2K). We validated the EBV RNA‐seq data in corresponding FFPE specimens with an independent method using digital EBER expression (NanoString Inc.) [49] and the two platforms showed significantly concordant results (Pearson correlation, *r* = 0.73; *p* < 0.001) (Figure S5S). EBER ISH and CD20 IHC were performed (Figure 2L), but showed no correlation (Figure S5T).

### 3.5 Epigenetic Reprogramming Targets TCR and PI3K Signaling in AITL

**FIGURE 3:**
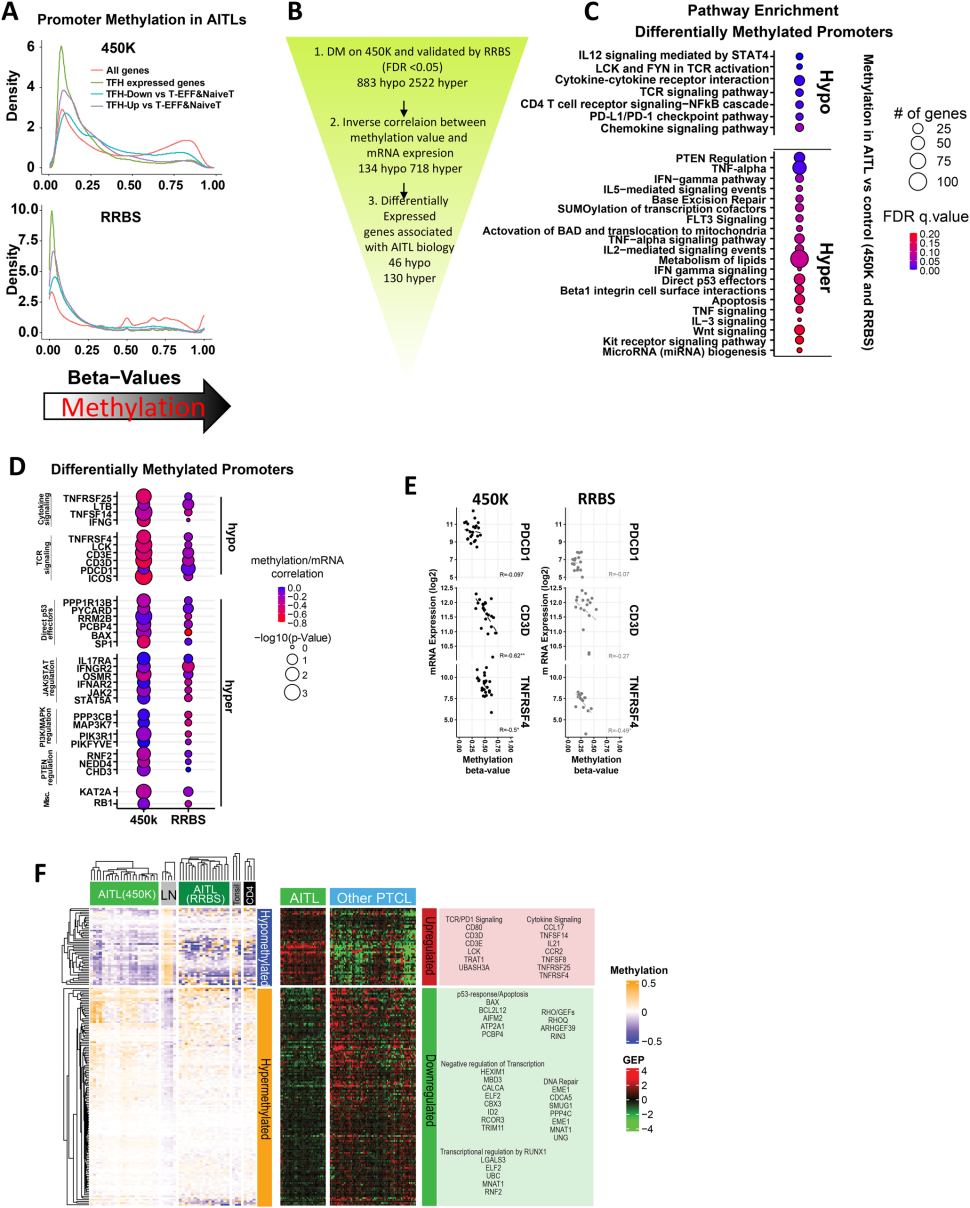
Methylation analysis with AITLs. (A) Distribution of promoter methylation levels of all genes and T_FH_ cell‐related gene sets in the AITL 450 K dataset (upper) and AITL RRBS dataset (lower). T_FH_‐expressed genes represent all genes that were expressed in the T_FH_ cells (FPKM > 2). T_FH_‐up/down versus T‐effector or naïve T‐cells were differentially expressed genes between the T_FH_ cells compared to T‐eff and naïve T cells. Beta‐values measure fractional methylation at CpG sites with 0 being fully unmethylated and 1 fully methylated. (B) Schematic of gene filtering strategy employed to identify disease‐relevant differentially methylated genes. (C) Enrichment analysis using concensus PathDB (http://cpdb.molgen.mpg.de/) of genes identified from Step 1 (B) whose promoter hypo‐ or hyper‐methylation status were consistent between platforms. (D) Selected genes whose promoter methylation levels were inversely correlated with mRNA expression and consistent between platforms that were identified from Step 2 of (B). (E) Inverse correlation between promoter methylation and mRNA expression for selected genes associated with **T**_FH_ biology. Significant correlations (*p < 0.05, **p < 0.01) are marked with asterisks. (F) Genes with differentially methylated promoters (i.e., AITL vs. controls, lymph nodes, CD4+ T‐cells) that showed an inverse correlation with mRNA expression. Selected genes for analysis included genes significantly associated with AITL (i.e., differential mRNA expressed between AITLs vs. other PTCLs, Step 3 of B). [Color figure can be viewed at wileyonlinelibrary.com]

We performed genome‐wide DNA methylation (5‐mC) analysis of 23 AITLs using 450 K BeadChip (Infinium) and cross‐validated biologically important genes in 17 other AITLs with RRBS data [25, 29]. Initial global methylation analysis in the two data sets showed less promoter methylation when assessing a T_FH_‐expressed gene set in both platforms compared to all promoters (Figure 3A). We employed a robust, multi‐step filtering process (Figure 3B) to identify genes with differential promoter methylation cross‐validated on both platforms, showing inverse correlation with corresponding mRNA, and were differentially expressed between AITLs and other PTCLs (Table S8). As shown in Figure 3C, the hypomethylated promoters included genes involved in T‐cell differentiation and activation, TCR‐NF‐κB and chemokine signaling, while hypermethylated promoters were enriched for genes involved in PTEN‐AKT signaling (negative regulators), p53 effectors, and apoptosis (Figure 3C). Genes with hypomethylated promoters and inverse correlation with mRNA expression included key genes associated with T_FH_ cell differentiation (*PD1, ICOS, TNFRSF4* (*OX40*)) and T‐cell activation (*CD3D*, *LCK, FYN*) and hypermethylated genes were negative regulators of the PI3K pathway (*PIK3R1*), or T‐cell activation (*SP1*) and effectors of p53, apoptotic regulators, and tumor suppressor genes (*BAX*, *RB1*) (Figure 3D,E), suggesting altered methylation in genes regulating T‐cell activation and TCR or PI3K pathway in AITL. The differentially methylated genes with promoter methylation negatively correlated with mRNA expression were also differentially expressed between AITLs and other PTCLs and mainly were associated with either upregulated TCR signaling, cytokine signaling, or downregulation of pro‐apoptotic factors or T‐cell differentiation transcription factors (Figure 3F).

### 3.6 AITL PDX Maintain Genetic Alterations in Subsequent Passages

**FIGURE 4:**
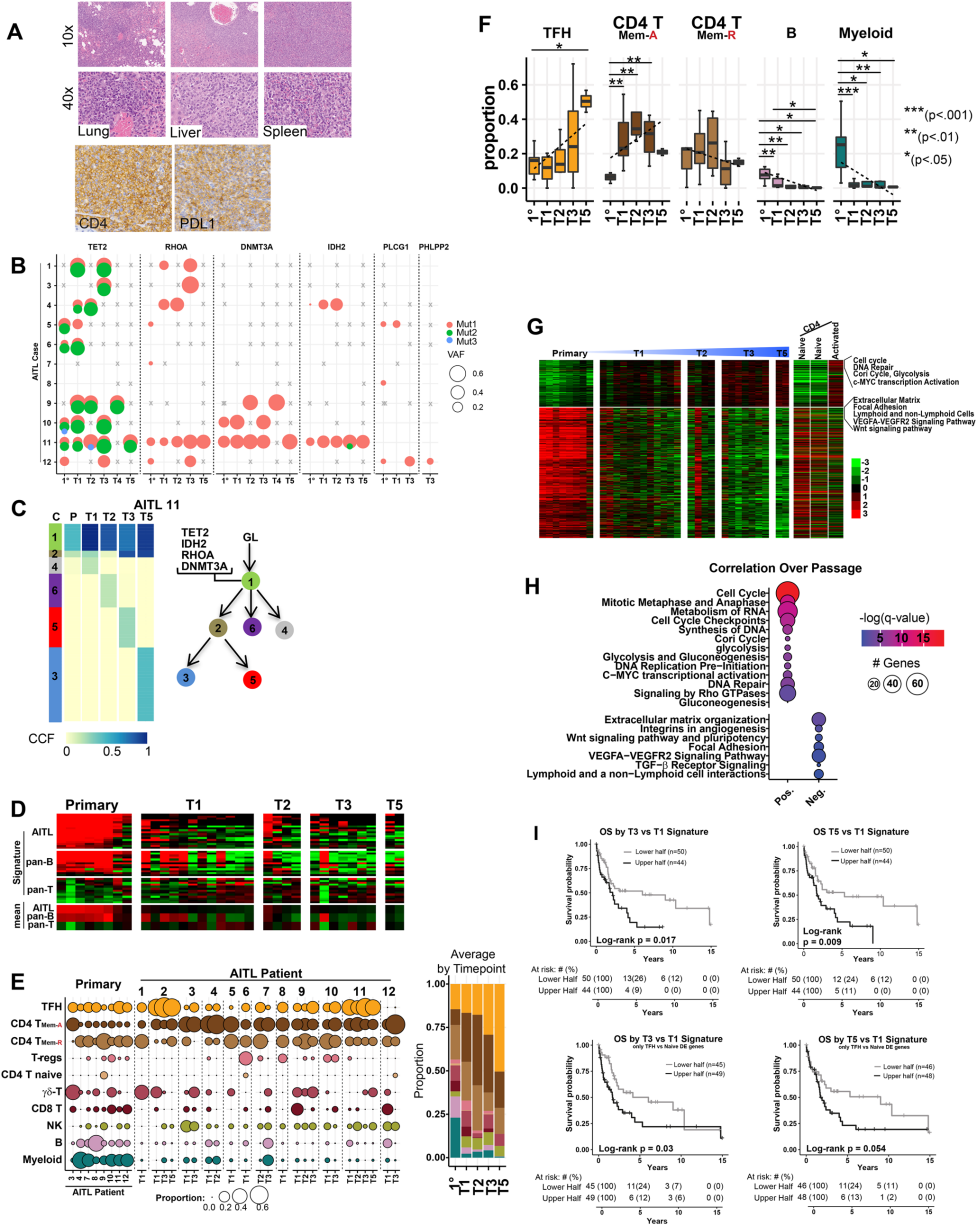
Molecular characterization of patient derived Xenograft (PDX) of AITLs. (A) H&E of mouse parenchymal organs (i.e., lung, liver, and spleens) from of NSG mouse implanted with a primary AITL biospecimen (second passage). Immunostains (×40) from spleens show that the majority of the tumors T‐cells have T_FH_ immunophenotype (CD4+ PDL1+) demonstrating infiltration of neoplastic cells in these organs. (B) Variant allele frequencies (VAF) of recurrent AITL mutations detected in PDX models over passage time. The size of the bubble corresponds to the VAF of the mutation, indicating higher VAF in secondary passages. Time points that are not available for each case are denoted by a gray x. (C) Distribution of Cancer Cell Fraction (CCF) values for mutations across various clusters and sub‐clonal evolutions in a representative Patient‐Derived Xenograft (PDX) model. Similar clusters were utilized to calculate a potential phylogenetic tree from the CCF values recorded in the data matrix (left). Using Revolver [50], phylogenetic trees illustrating the clonal evolution of each variant were constructed (right). Circles in the trees represent clusters with and without driver gene mutations. “GL” denotes germline, C: Cluster, P: Primary; and T1, T2, T3, and T5 for Passages 1, 2, 3, and 5, respectively. (D) Evaluation of the previously defined AITL diagnostic signatures (mean expression levels) pan‐B and pan‐T‐cell signatures in the primary and secondary PDX passages demonstrating that neoplastic content is increasing, while TME is decreasing in secondary passages. (E) Cellular subset composition estimation of RNA‐seq data by CIBERSORT. The bar plot along the right represents the average proportion based on all samples in the time point. (F) Noted cell subsets estimated by CIBERSORT over passages. The dashed line in the plot represents a linear regression trendline. *p*‐values were calculated by Wilcoxon rank‐sum test. (G) Upregulated and downregulated genes observed in secondary passages over time (T1–T5). Pathways that are significantly affected by up‐ or down‐regulated genes are noted. (H) Pathway enrichment analysis of genes that were significantly up or down‐regulated over passage time. (I) Association of gene signatures identified in secondary PDX passages with OS in AITLs. Differential expression of signatures associated with late PDX passage (T5 vs. T1 or T3 vs. T1), upper left and right or T5 versus T1 or T3 versus T1 were evaluated in AITL cohort with GEP data. AITL cases were divided into the upper half and lower half based upon expression of the signature and associated with OS. Cases were also evaluated using T_FH_ related genes only (i.e., genes differentially expressed between T_FH_ and naïve T‐cells (lower left & right). [Color figure can be viewed at wileyonlinelibrary.com]

We generated AITL PDXs (*n* = 12) and propagated them for consecutive passages (T_1_ to T_5_) and observed systemic dissemination of AITL cells to mouse parenchymal organs (liver, lung) and lymphoid tissues (i.e., spleen) and post‐transplant mouse tissues were infiltrated by a sizable percentage of human CD4+ T‐cells (Figure 4A). Genetic analysis of the primary lesions (1^0^) was cross‐validated in several PDX lines post‐transplant (T_1_–T_5_), demonstrating genomic signatures representative of the donor implants [15] (Figure 4B) [51]. In addition, increasing variant allele frequency (VAF) of recurrent mutations, including*TET2, RHOA* ^*G17V*^, *DNMT3A, IDH2* ^*R172*^, and *PLCγ1* observed in several models over passage, indicate clonal expansion or tumor cell enrichment in subsequent passages (Figure 4B). AITL cases with two *TET2* mutations, likely bi‐allelic, propagated with equal VAF over several passages, indicating selective pressure to maintain inactivated TET2. However, AITL with a single *TET2* mutation evolved with *RHOA* ^*G17V*^ and *PLC*γ1 mutation over passages, and a *PHLPP2* non‐sense mutation was identified in a T_3_, suggesting sub‐clonal presence and possible selective pressure for PI3K activation for clonal expansion (Figure 4B). *RHOA* ^*G17V*^ mutation in two AITL cases did not persist post‐transplant, indicating they were sub‐clonally. We also performed integration of the primary and PDX genetic data for clonal evolution analysis (see Supporting Information methods for details), which suggested *TET2* loss was the primary driver for clonal evolution in a majority of the AITL PDXs, while *RHOA* ^*G17V*^ was a secondary change that may vary during clonal evolution, and several other potential driver genes, including *DNMT3A, IDH2* ^*R172*^, *PLCG1/2*, or *TET3*, generally expand during passage (Figures 4C and S6A,B).

GEP analysis showed that the primary specimen had high expression of the AITL signature enriched with tumor microenvironment (TME) elements, including B‐cell and dendritic cells that progressively decrease with passaging in mice (T_1_ to T_5_) (Figure 4D). CIBERSORT analysis identified enrichment of **T**_FH_ and CD4 T‐cell memory (activated), while B‐cells and dendritic cells progressively decreased (Figure 4E,F), indicating selection of tumor T‐cell clones that were less TME‐dependent.

Transcriptomic analysis identified that genes involved in the cell cycle, mitosis, and metabolic reprogramming (i.e., glycolysis, gluconeogenesis), and MYC activation increased over passages (i.e., positive correlation, T_1_–T_5_), while those involved in extracellular matrix organization and lymphoid/non‐lymphoid cell interaction, and likely microenvironmental in origin, decreased over time, and these enriched gene signatures were preserved in in vitro activated CD4 T‐cells (Figure 4G,H). Next, we assessed whether gene signatures related to late PDX passages predict patient survival and observed differential GEP between (T_5_ vs. T_1_) or (T_3_ vs. T_1_) were able to predict OS in the AITL cases (Figure 4I, upper). Of these gene signatures, we selected a subset of genes expressed in T_FH_ cells, and this subset also demonstrated prognostic significance, suggesting that PDX models can be relevant biological and prognostic models (Figure 4I, lower).

### 3.7 Pathogenic Role of PI3K Alterations

**FIGURE 5:**
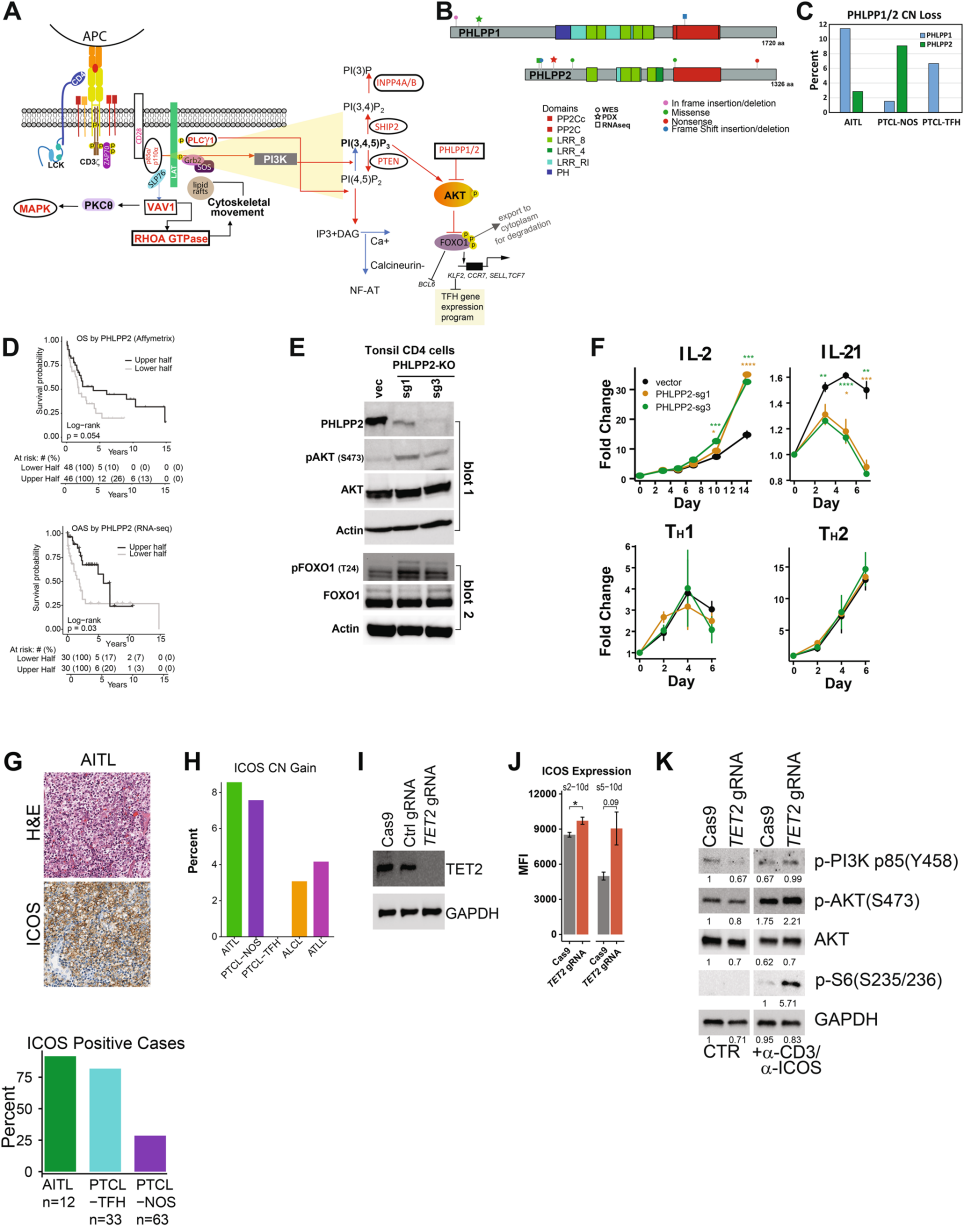
Abnormalities associated with PI3K activation in AITL. (A) The schematic of PI3K pathway and mutant genes identified by WES in this AITL study are indicated in red letters. (B) Schematics of *PHLPP1 and PHLPP2* mutation spectrum in AITL. (C) Frequency of *PHLPP1* and *PHLPP2* DNA copy number loss in AITL compared to PTCL‐NOS and PTCL‐T_FH_. (D) Association of OS with mRNA expression level of *PHLPP2* using two different platform (i.e., HG‐U133plus2 array [left panels] or RNA‐seq [right panels]) showing inferior OS associated with low *PHLPP2* expression. (E) Western blot for indicated proteins (PHLPP2, AKT, FOXO1) in PHLPP2^−/−^ CD4+ T‐cells showed down‐regulation of PHLPP2, but higher pAKT and pFOXO1 suggesting activation of AKT‐FOXO1 signaling. β‐actin was used as a control for protein lysate. (F) Cell proliferation curve of CD4+ T‐cells with/without *PHLPP2* knock‐out cultured in standard culture conditions containing IL‐2, IL‐21, T_H_1 polarization conditions, or T_H_2 polarization conditions. Gold asterisk denotes significance for vector compared *PHLPP2*‐sg‐1 and green asterisks, vector compared to *PHLPP2*‐sg‐3 (*p* < 0.05*, *p* < 0.01**, *p* < 0.001***, *p* < 0.0001****). (G) H&E and ICOS IHC in a representative AITL case (×20), upper panel. Bar plot comparing the frequency of ICOS positivity in AITL, PTCL‐T_FH_, and PTCL‐NOS, lower panel. (H) Frequency of 2q33.2 CN gain encompassing ICOS gene in AITL and other PTCL using data previously published (Heavican et al., Blood 2019). (I) Western blot for TET2 expression in *TET2* Knock‐out CD4+ T‐cells compared to WT cells confirms *TET2* Knock‐out. (J) ICOS expression assessed by flow cytometry in WT and TET2 knock‐out CD4 T cells from three separate donors examined on the 10th day (10d) after the second (S2) or fifth (S5) round of α‐CD3/CD28 beads stimulation after *TET2* knock‐out. *p < 0.05, *t*‐test. (K) Western blot examination of PI3K/AKT/mTOR signaling activation with 2 days α‐CD3/α‐ICOS stimulation in WT and TET2 knock‐out CD4 T cells. Densitometry values for the bands are denoted below and show values for a representative experiment of two independent experiments. [Color figure can be viewed at wileyonlinelibrary.com]

Several non‐overlapping mutations, promoter hypermethylation, or copy number abnormalities [22] in different regulatory subunits of PI(3)K suggest an aberrant PI3K pathway in AITLs, which was also supported by GEP analysis (Figure 5A). Of interest were abnormalities affecting phosphatases (*PHLPP1/2*) due to the acquisition of either a truncating mutation and/or g‐CN loss (20% AITLs) resulting in reduced mRNA expression or loss‐of‐function (Figure 5B,C). *PHLPP2* mRNA expression correlated with OS, with low mRNA expression showing poor OS in two AITL cohorts (Figure 5D).

To demonstrate the role of PHLPP2, we performed knock‐out of *PHLPP2* using CRISPR gRNA in peripheral CD4+ T‐cells from three healthy donors. *PHLPP2* knock‐out CD4+ T‐cells had higher levels of p‐AKT and p‐FOXO1, resulting in inactivation of FOXO1 signaling, thus promoting T_FH_ differentiation [52] or reducing the apoptotic process by lowering BIM expression [53] (Figure 5E). *PHLPP2* knock‐out CD4 + T‐cells showed proliferative advantage in vitro in media including αCD3/αCD28 and IL2, a cytokine negative regulator of T_FH_ differentiation [54] (Figure 5F), but not when cultured with IL21, a cytokine promoting T_FH_ differentiation [55] (Figure 5F). Upon culturing *PHLPP2* knock‐out CD4+ T cells in T_H_1 and T_H_2 polarizing conditions, the cells survived in T_H_2, but not in T_H_1 conditions (Figure 5F). While *PHLPP2* knock‐out CD4+ T‐cells had more cells in S‐phase 5 days post‐stimulation with IL‐2 and α‐CD3/anti‐CD28, there was no significant difference in apoptosis (Figure S7A,B). Notably, *PHLPP2* knock‐out led to PI3K pathway activation (Figure 5E) but had no effect on proximal TCR‐mediated signaling (Figure S7C).

Several lines of evidence, including GEP, IHC, CN gain, and methylation analysis, suggest higher ICOS expression in AITL. ICOS is a key mediator of PI3K activation in T_FH_ cell [56], and is part of the diagnostic criteria for AITL, with ICOS positivity significantly associated with AITL (95%) vs. other PTCL subtypes (0%–40%) [57] (Figure 5G). ICOS mRNA was expressed at significantly higher levels in AITL compared to other PTCLs. The ICOS promoter was often hypomethylated, and recurrent copy number gains (8%) were observed (Figures 5H and S7D,E). Since ICOS expression and *TET2* alterations are key features in AITL, we examined whether TET2 may regulate ICOS expression. To test this association, we modified normal CD4+ T‐cells by CRISPR to delete *TET2* (Figure 5I) and showed a modest increase in ICOS expression in *TET2* ^*−/−*^ CD4+ T‐cells (Figure 5J). When stimulated with α‐ICOS, these modified T‐cells showed increased phosphorylation of AKT and p70 S6 kinase, indicating that the ICOS with ligand interaction translated to higher AKT‐mTORC1 activation, thus associating *TET2* loss with enhanced PI3K/AKT/mTOR activation through upregulation of ICOS (Figure 5K). Overall, these findings further corroborate that hyper‐PI3K activation, either mediated by functional loss of negative regulators or indirectly by functional gain of positive regulators, is a major event in AITL.

## 4 Discussion

We used a multi‐omics approach, integrated with clinicopathological and molecular characteristics to establish the genetic landscape in AITL. A major challenge in deciphering the genetic landscape is low tumor content often associated with the AITL biospecimens, thus we prioritized cases with higher sequencing depth and tumor‐normal pairs. We discovered potential therapeutic targets through integrative multi‐omics analysis [14, 58, 59] and extended earlier findings that epigenomic dysregulation and aberrant TCR activation are the two major genetic events in AITL [13, 14, 16, 18]. DNA methylome deregulation is a principal pathogenetic feature due to frequent mutations affecting *TET2*, *IDH2*, and *DNMT3A*, and although infrequently, *TET3* was also mutated with *TET2* mutation, probably producing cooperative interactions in AITL pathogenesis. Approximately 55% AITLs carried two *TET2* mutations per case, and 7/9 AITL‐PDX models with *TET2* mutations had two mutations (i.e., likely bi‐allelic) propagated through subsequent passages suggesting bi‐allelic *TET2* loss is important for lymphomagenesis. In contrast, bi‐allelic *DNMT3A* mutation was not frequent. In a subset of AITLs, *TET2* or *DNMT3A* have higher‐than‐expected VAF relative to other mutations or tumor content and are possible founder mutations present in hematopoietic stem/progenitor (HSPC) cells [16, 17, 32, 58, 60, 61, 62, 63]. These mutations significantly co‐occur in AITL, but they are mutually exclusive in clonal hematopoiesis of indeterminate potential (CHIP) or myeloid diseases [64]. We performed sequencing analysis of the sorted tumor CD3^+^PD1^+^ cells, established clonal enrichment in the sorted fraction using TCRγ assessment, and WES results were concordant with their corresponding tissue biopsies. Three cases showed *TET2* or *IDH2* mutation in sorted cells that were undetected in the whole‐tissue section, likely due to low tumor content and sub‐clonal mutation. Consistent with previous studies [38, 39], *TET2* and *DNMT3A* mutations were observed in non‐tumor cells including myeloid or B‐cells, suggesting early founding mutation in a progenitor cell in a subset of cases, but other mutations like *IDH2, RHOA*, and *CD28* appeared to be tumor‐specific. Interestingly, *TET3* mutation frequency was relatively higher in the sorted neoplastic cell group and some mutations were tumor‐specific and likely subclonal. TET2 and DNMT3A have opposite functions and their effect on methylation dysregulation is likely complex and understanding target genes affected by methylation alterations due to single or combined TET2 and DNMT3A mutations is important and it will be critical to elucidate their individual and cooperative role in AITL tumorigenesis. TET2 or DNMT3A deficiency in HSPCs may favor a distinctive tumor milieu, but the functional effects of their loss in the TME of AITL needsx further exploration. Our analysis suggests that such AITLs tend to skew B‐cell content, with increased myeloid content in the TME, indicating functional impacts on the TME. The inverse correlation between B‐cell content and myeloid content in the TME was validated by representative IHC markers and their respective abundance showed significant association with clinical outcome [17, 34]. EBV transcripts were present in 70% AITLs, as noted in earlier studies [47], but were not correlated with B‐cell content, indicating that the extent of B‐cell infiltration may be EBV independent. In addition to aberrant DNA methylation, histone methylation would be expected to be perturbed in at least 25% AITLs due to *IDH2* ^*R172*^ generated 2‐HG resulting in impaired function of a large group of histone lysine demethylases. While genes regulating histone methylation (*KMT2A/2C/2D/5A*) were mutated in ~6% AITLs, *KMT2D* mutations were associated with cases lacking *TET2* mutation. The complex and extensive epigenetic alterations likely account for the more frequent and better response of this disease to drugs that modify the epigenome such as 5‐Aza, HDACi, and EZH1/2i [65].

Acquisition of mutations regulating TCR signaling is likely beneficial to lymphoma evolution, as most of these recurrent aberrations (mutations, fusions, and/or differential methylation) showed gain‐of‐function and may result in enhanced TCR activation [16, 35, 36, 41]. The role of RHOA^G17V^ is complex and may require prior TET2 loss to promote lymphomagenesis. *TET2* and *RHOA* ^*G17V*^ alterations in murine studies suggest a synergistic effect leading to atypical CD4+ T‐cell proliferation, TCR activation, and an imbalance in T_H_ differentiation via ICOS upregulation and inactivation of FOXO1 [32, 66]. RHOA^G17V^ recruits VAV1, promoting its activation and inducing active TCR signaling [66]. Impaired RHOA function may lead to enhanced PI3K and RAC activation [67]. The presumed dominant negative RHOA^G17V^ mutation is particularly intriguing, as it is distinctively associated with T_FH_‐associated PTCL. Other loss or gain‐of‐function mutations in RHOA are seen in other PTCLs [68], but not in AITL. That RHOA^G17V^ co‐occurs with CD28 or PLCγ1 [16, 19, 20, 21] mutation may also indicate non‐overlapping roles in TCR activation. Indeed, global methylation analysis indicated recurrent hypomethylation of genes leading to activation of TCR signaling. In contrast, RHOA WT cases showed more frequent JAK–STAT pathway mutations suggesting alternative oncogenic mechanisms in such cases.

One of the novel findings was the frequent alterations targeting PI3K/AKT activation in AITL with significant implications in therapeutics. PI3K‐pathway regulation is perturbed by several mechanisms, including ICOS upregulation [56] due to genetic or epigenetic alterations, acquisition of somatic mutations in key regulatory genes (e.g., *PI3K3R1, PI3KR5*, and *PIK3CA*) or mutation, copy number loss, or promoter methylation in phosphatases (*PHLPP2* or *PHLPP1*). Interestingly, low *PHLPP*2 expression was associated with inferior outcomes in AITLs. Both PHLPP1 and PHLPP2 can dephosphorylate AKT and RAF1, inducing apoptosis and inhibition of migration [69, 70]. Our in vitro experimental studies of *PHLPP2* loss in CD4+ T‐cells show a growth advantage in IL2 culture, but not in T_FH_‐promoting conditions. *PHLPP2* knock‐out led to activation of the AKT‐PI3K pathway and phosphorylation (T24) of the transcription factor FOXO1, which is associated with its nuclear export and inhibition of its transcriptional activities [71], and FOXO1 inhibition promotes T_FH_ differentiation [52]. No significant change in TCR signaling with *PHLPP2* loss suggests that PI3K activation may complement TCR activating mutations in AITL. One significant observation was that TET2 loss can induce ICOS expression in CD4+ T‐cells and can result in PI3K activation. This is consistent with the frequent high ICOS expression in AITL and many ICOSL‐expressing cells, including B‐cells in the TME that would provide the necessary stimulus. These findings indicate that PI3K signaling in AITL may be frequently and often cooperatively activated by several interrelated mechanisms, such as TCR signaling pathway activation, *RHOA* ^*G17V*^ mutation, and *TET2* loss. The functional genomics via RNA‐seq analysis validated these dysregulated oncogenic pathways. This is intriguing, as constitutive PI3K activation is targetable with several currently available clinical‐grade inhibitors [72] and the phase 2 PRIMO trial (NCT03372057) found that relapsed/refractory AITLs have a 62.2% overall response rate to duvelisib treatment, higher than the other PTCLs [73].

PDXs are presumed to better represent the genomic features of primary tumors than cell lines. The genetic alterations in 12 PDX specimens suggest persistence of *TET2* and *DNMT3A* loss during tumor passaging, but *RHOA* ^*G17V*^ showed more variations with disappearance in some PDX models and re‐emergence that follows TET2 loss in some after several passages, indicating more dynamic clonal selection. Biallelic *TET2‐deficient* clones were selected in the PDX models. PDX neoplastic CD4+ T‐cells clonal expansion is associated with a memory‐like GEP signature, enriched with T_FH_‐like gene expression. GEP signatures derived from PDX tumors showed higher metabolic activation and proliferative signatures compared to primary tumor cells. Importantly, the transcriptomic signature enriched in PDXs was associated with OS, suggesting the signature represents a more aggressive and TME‐independent clone and may be associated with therapy resistance and recurrence.

In summary, we defined the genomic landscape for AITL, which is characterized by epigenetic alterations, TCR signaling, and PI3K/AKT dysregulation that may be amenable for therapeutic targeting. Several abnormalities were associated with inferior clinical outcomes, including triple mutation of *TET2*, *IDH2*, and *DNMT3A*, and *CD28* mutation/fusion. As the cases in this study were collected over a long timespan and from multiple institutions, future validation of the prognostic findings in a larger cohort with more uniform management and trials with novel or targeted therapies would be worthwhile. Epigenetic targeting agents, PI3K inhibitors [74], SRC kinase inhibitors, and RAC/RAS inhibitors or potential combinations are already under investigation and may be studied in future clinical trials. In addition, as single‐cell‐based sequencing becomes more affordable, in‐depth characterization of the genomic architecture of AITL, especially the TET2 and DNMT3A relationship, will become possible.

## Author Contributions

A.B., J.I., and W.C.C. designed and performed the research. A.B., J.I., W.Z., S.S., R.A.S., Q.G., W.G.L., R.Y., X.L., S.M., R.S.C., and M.A.S. performed experiments and/or analyzed and/or interpreted the data. All other authors provided materials, conducted the pathology review, and/or contributed clinical data. All authors edited and approved the manuscript. J.I. and A.B. wrote the initial draft. J.I. and W.C.C. finalized the manuscript.

## Conflicts of Interest

The authors declare no conflicts of interest.

## Supporting information


**Data S1.** Supporting Information.

## Data Availability

Data will be deposited in the GDC database upon acceptance for publication or available by contacting the authors.
