# Supplementary material for: Integrative Genomic and Transcriptomic Analysis Reveals Targetable Vulnerabilities in Angioimmunoblastic T‐Cell Lymphoma
Source: Am J Hematol. 2025 Jun 13;100(9):1486–501. doi: 10.1002/ajh.27736 (PMC12326222; doi:10.1002/ajh.27736)
Supplement: Supplementary file 1 — Data S1. Supporting Information. [file AJH-100-1486-s001.zip › AITL.supplemental.figures.v13.FINAL.AJH.pdf]

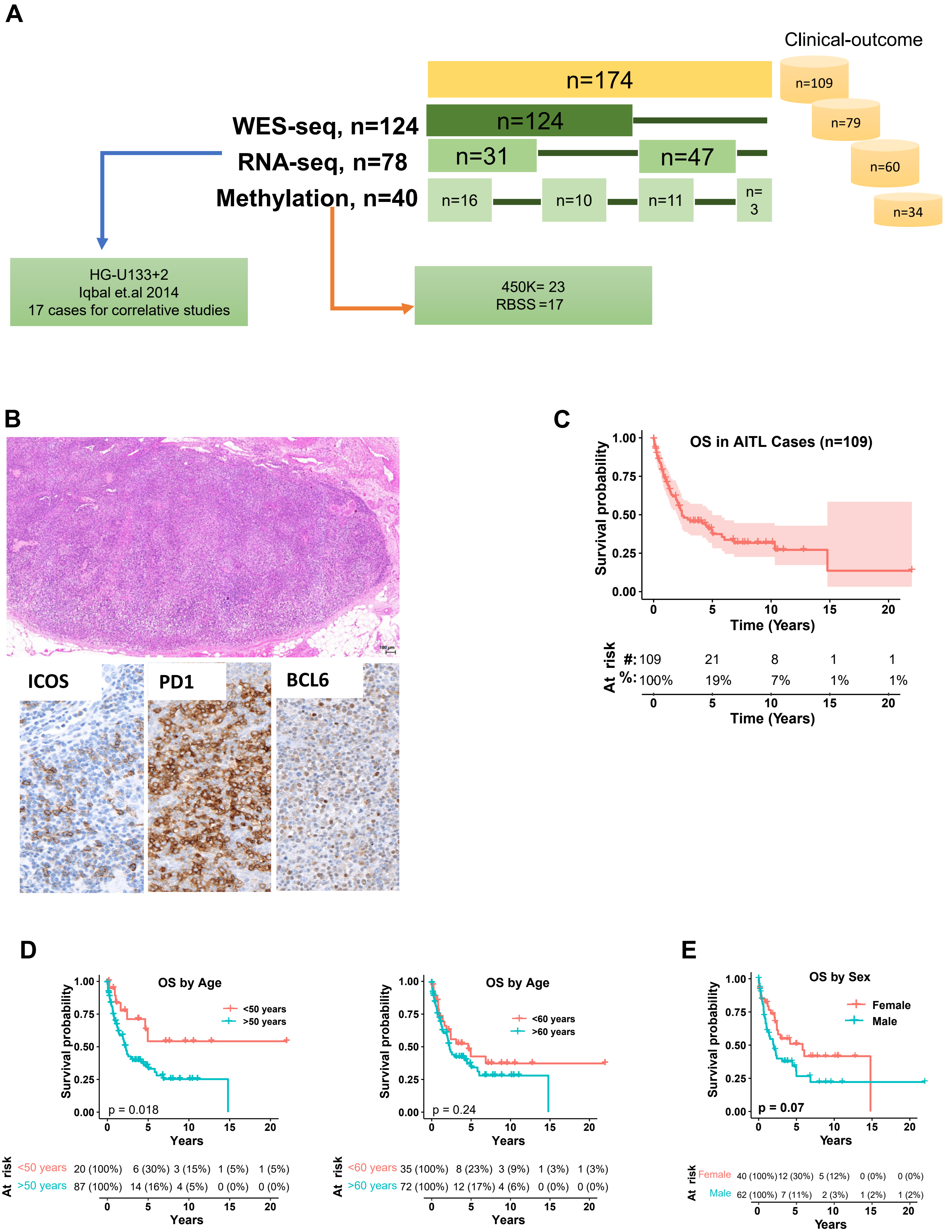


**Figure-S1.** A. Schematic of study cases and the data available. B. Representative figure of AITL case H&E (upper) and the noted immunostains (lower). Kaplan-Meier curves of overall survival of AITLs in the study with available outcome data (C) or by age (D) or sex (E).

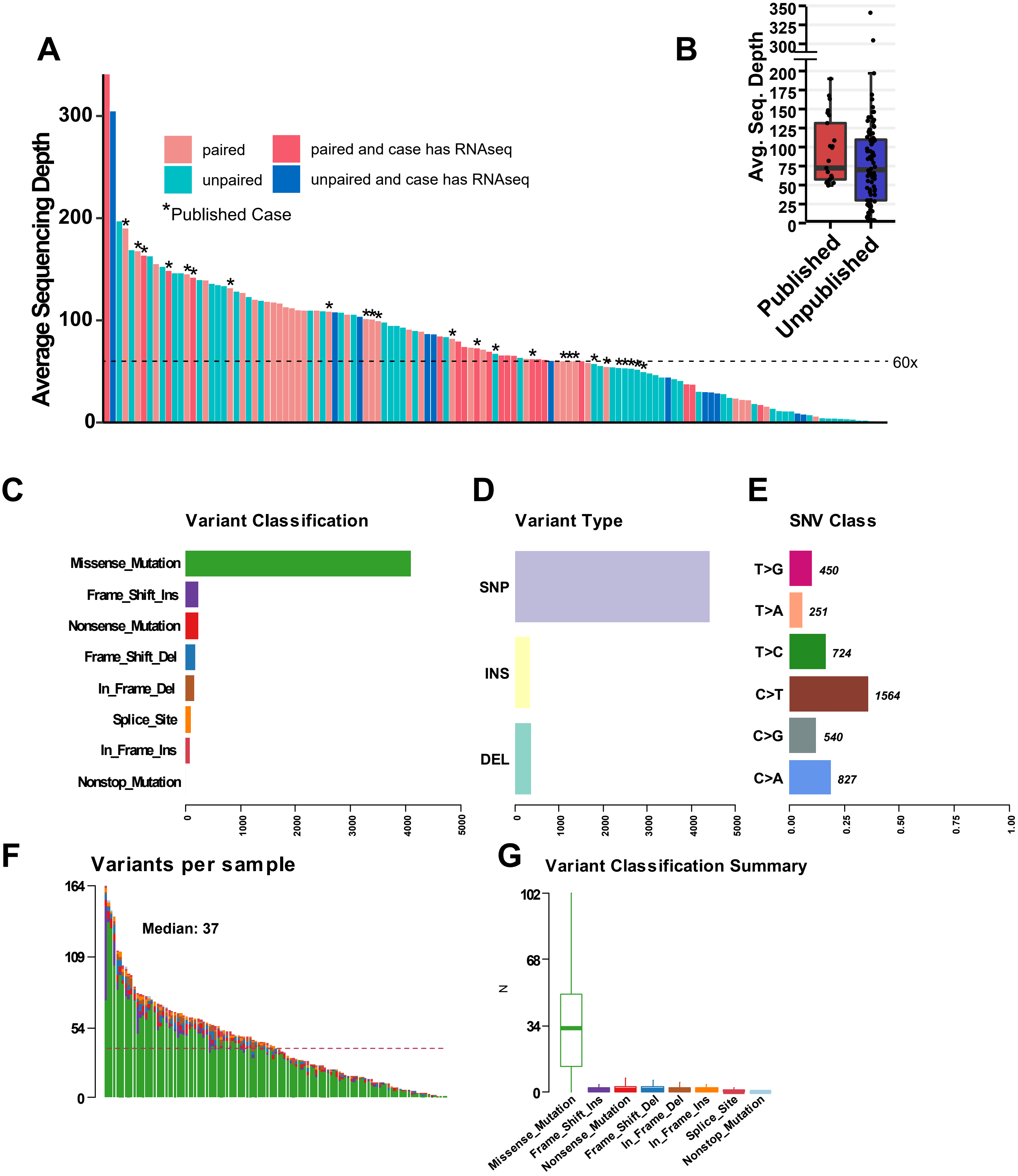


**Figure S2. Summary sequencing depth and variants detected by WES in AITL cases.** A. Average whole exome sequencing depth of all cases in study. Previously published cases and cases with available germline (paired) are noted. B. Boxplot of average sequencing depth in new and previously published cases. C. Bar graph of the number of mutations detected for each variant class. D. Bar graph of the number mutations detected for the noted variant type. E. The proportion of variants detected by base change. F. Summary of the mutation detected in each sample. G. Boxplot of the mutations/sample for the noted variant classifications. Colors in F-G correspond to the variant classification colors noted in C.

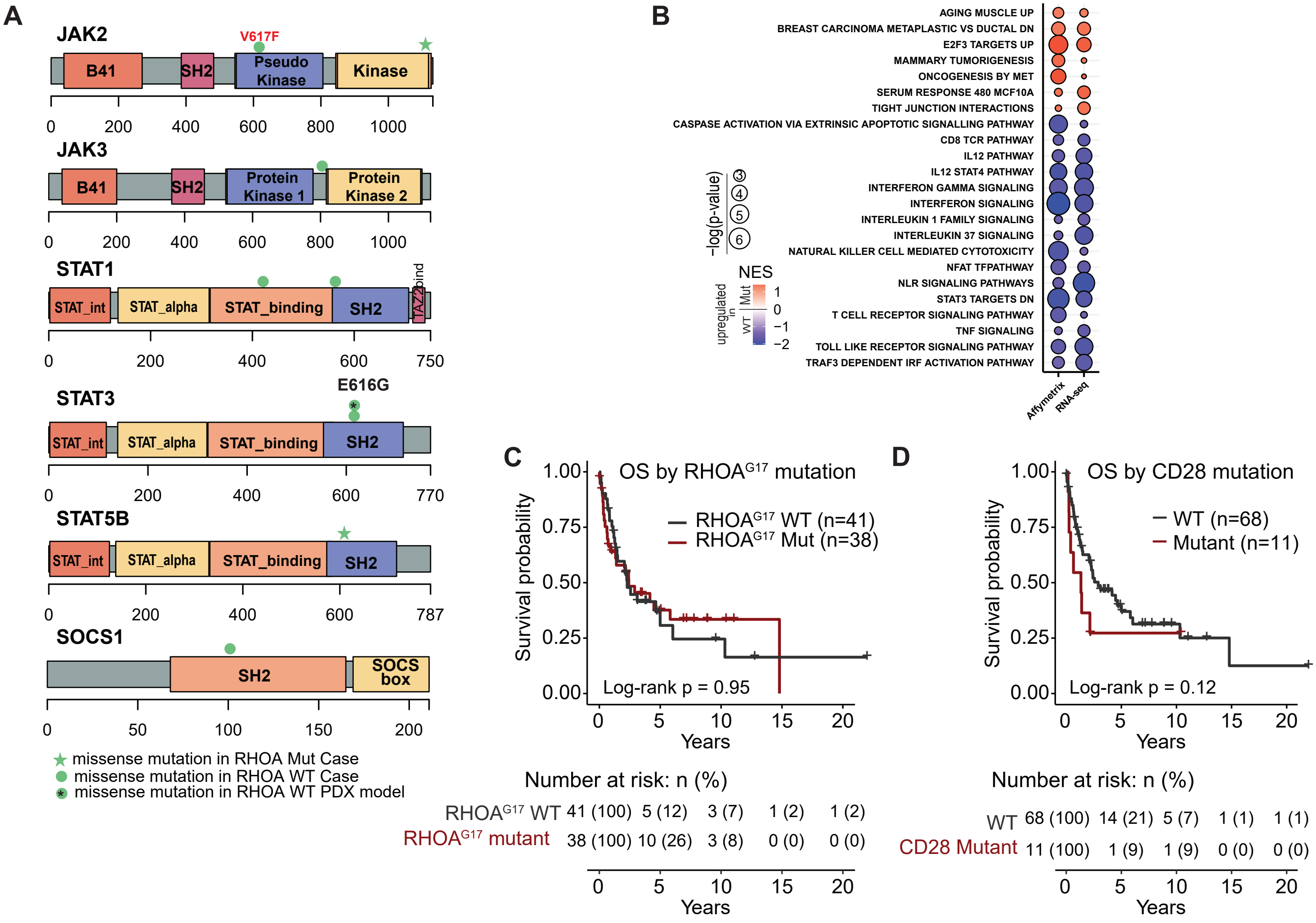


**Figure S3.** A. Protein schematic of mutation location in JAKs, STATs, and SOCS1. B. Bubble plot of gene set enrichment analysis comparing RHOA WT and RHOA mutant in Affymetrix array data set and the RNA-seq dataset. Select pathway that were less than p<0.1 in both datasets are shown. Kaplan-meier curve comparing OS in TET2-mutant AITL cases by RHOA status (C) and CD28 (D).

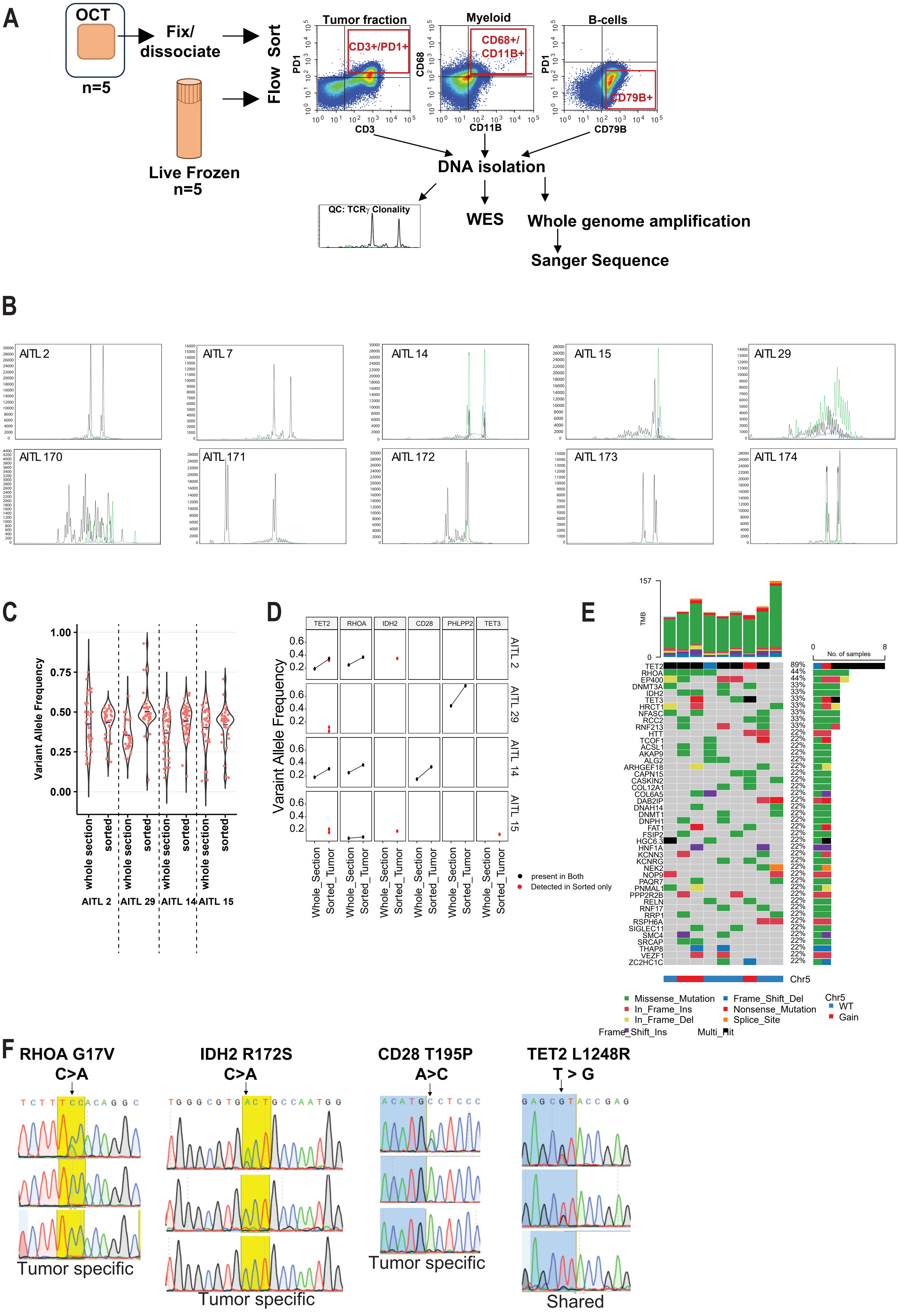


**Figure-S4.** A. Scematic of AITL sorting strategy. B. Assessment of TCR-gamma chain clonality in PD1+/CD3+ sorted AITLs by capillary electrophoresis. C. Comparison of variant allele frequencies for mutations detected in the DNA isolated from a whole section versus DNA isolated versus DNA isolated from CD3+/PD1+ tumor sorted cells. D. Comparison of variant allele frequencies mutatations in the noted genes in the DNA isolated from a whole section versus DNA isolated from CD3+/PD1+ tumor sorted cells. E. Oncoplot of most common mutations detected in sorted CD3+/PD1+ tumor cells. F. Example Sanger sequencing chromatograms for tumor specific or shared mutations from AITL-7.

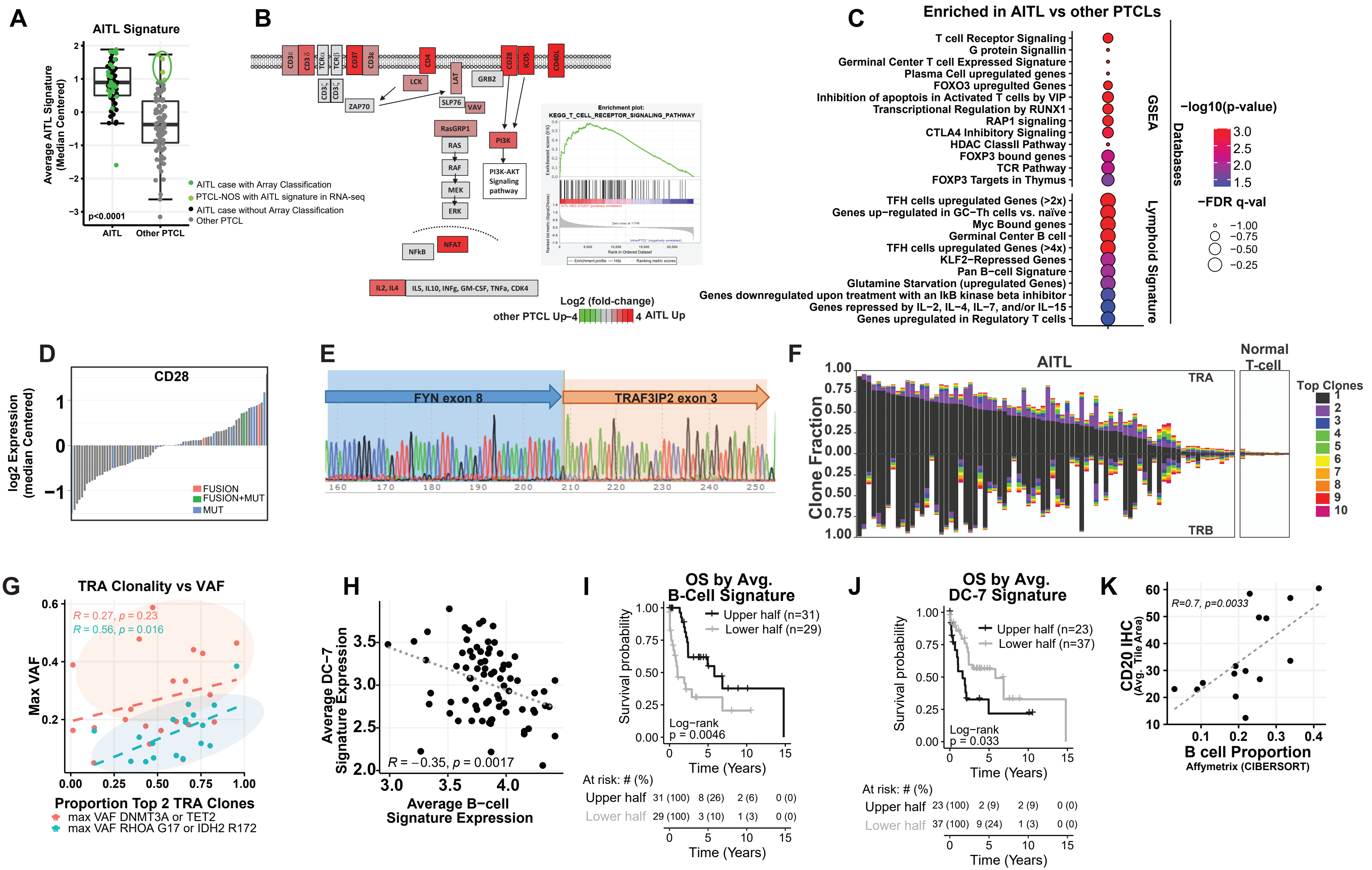


**Figure S5.** A, Mean-expression levels of AITL-diagnostic signature genes assessed through RNA-seq data is compared between AITL vs other PTCL. The p value was calculated by Student's T-test. The 4 PTCL-NOS cases with high AITL signature are circled in green. B. The schematic of T-cell receptor (TCR) pathway enriched in AITL compared to the other PTCLs. Upregulated genes associated with T-cell activation identified via GSEA analysis (inset) and shown in red using KEGG pathway graphics. C. List of top enriched gene-signatures in AITLs compared to other PTCLs identified using GSEA utilizing lymphoid signature databases (Shaffer et.al Immunol Rev. 2006). D. Association of *CD28* mutation and fusion status with mRNA expression with denoted in color. AITLs with CD28 alterations trended to have higher mRNA expression. E. Sanger sequencing confirmation of the fusion from the FYN::TRAF3IP2 fusion positive case. F. Contribution of TRA CDR3 α and TRB CDR3β sequences to the TCR repertoire in 78 AITLs and 10 normal T-cell samples. G. Scatter plot illustrating the VAF derived from WES data for tumor-specific variants (*RHOA*$^{G17V}$/*IDH2*$^{R172}$) and tumor+ tumor milieu variants (*TET2/DNMT3A*), in relation to the TCR-α clonal fraction (top 2 TCR-α clones) estimated by RNA-seq. The correlation coefficients, calculated using the Pearson method, are depicted, and the dashed line represents the linear regression trend. The data shows a significant correlation between tumor-specific VAF and TCR-α clonal assessment, while no significant correlation is observed with T*ET2/DNMT3A.* H. Scatter plot demonstrating Inverse association of dendritic-cell mRNA signature (DC-7) with the B-cell mRNA signature (estimated by CIBERSORT analysis) utilizing RNA-seq dataset (n=78). The data shows a significant inverse correlation (P<0.001) between the two signatures. The correlation coefficient was calculated by Spearman correlation and the dashed line represents the linear regression trendline. I. Association of OS with B-cell Signature expression. 78 AITL cases with RNA-seq were divided in two halves (upper vs lower half by B-cell Signature expression). AITL with higher B-cell mRNA signature are associated with good prognosis (p=0.0046). J. Association of OS in AITLs with DC-7 mRNA signature (average expression). AITL cases with RNA-seq were divided in two 2 halves based on mRNA expression and associated with OS. AITLs with high DC-7 mRNA signature were associated with poor prognosis (p=0.033). K. Association of CD20 IHC vs B-cell content estimated by CIBERSORT using RNA-seq. CD20 immunostains were quantified by QuPath software. There was a significant association between CD20 protein expression and B-cell content in AITLs (R=0.7 p=0.0033). Correlation coefficients were calculated by spearman correlation and the dashed line represents the linear regression trendline.

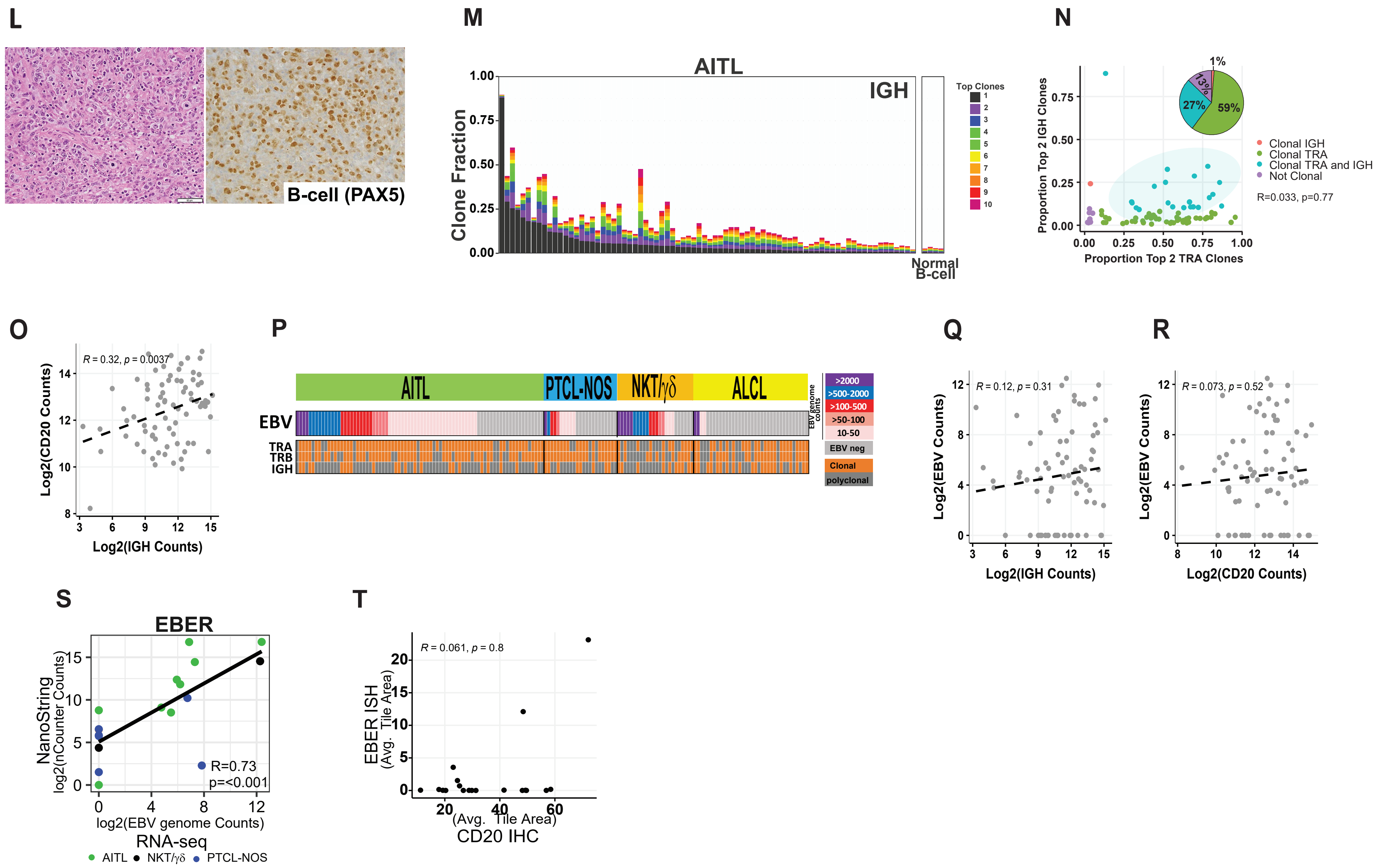


**Figure-S5 continued** L. H&E and PAX5 immunostain in an AITL case with B cell proliferation. M. Contribution of IGH CDR3 sequences to the B-cell repertoire in 78 AITLs and 4 normal B-cell samples. N. Scatterplot of the top 2 TRA clones and the top 2 IHG clones. Color specifies cases that are considered clonal and the inlayed pie graph depicts the proportion of the cases that are clonal. O. Comparison of IGH counts to CD20 counts. P. Identification of EBV transcript from RNA-seq in AITL and other PTCLs. Comparison of EBV transcript with T*CR-α*, *TCR-β* and *IGH* clonality assessed by RNA-seq. Read counts normalized to $10^7$ reads. Q. Comparison of IGH counts to EBV counts. R. Comparison of CD20 counts to EBV counts. S. Validation of EBV transcripts identified by RNA-seq and EBER expression measured by nCounter$^{TM}$ in cases profiled by both methods. T. Association of EBER ISH and CD20 IHC indicating no association between B-cell and EBV content. For O and Q-S correlation coefficients were calculated by Pearson correlation and for T by Spearman correlation. The line represents the linear regression trendline.

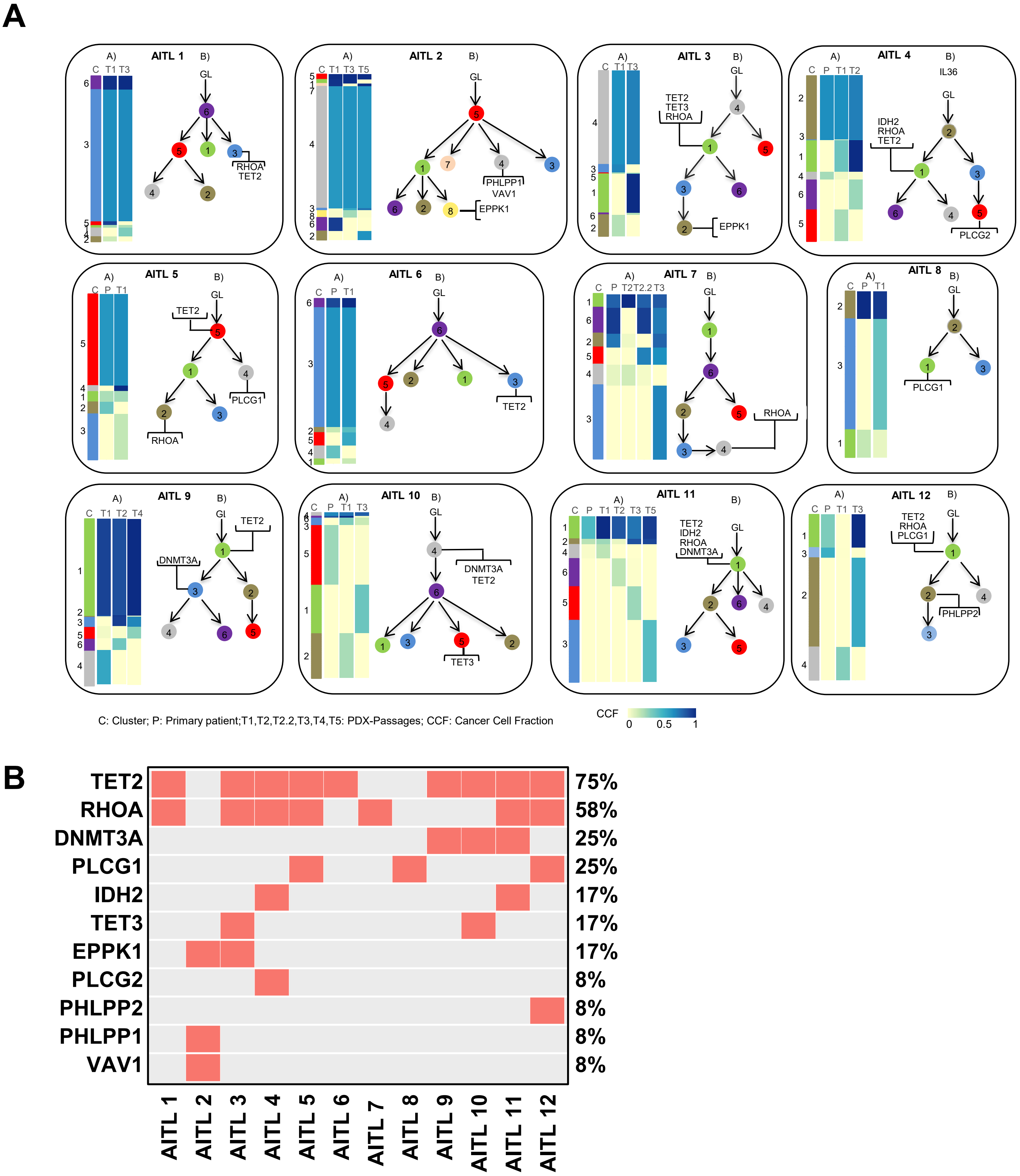


**Figure S6. Tumor evolution analysis in AIT**L A) Left.:Plot represents the distribution of mutations (Cancer Cell Fraction (CCF)) values in various clusuters and sub-clonal evolutions. The similar cluster are are used to compute the possible phylogenetic trees from the CCF values reported in the data matrix Right: Phylogenetic trees were plotted to show the clonal evolution of each variant using Revolver. The circles denote the clusters with and without driver gene mutations. B. Occurrence of most frequently mutated driver genes across individual patients

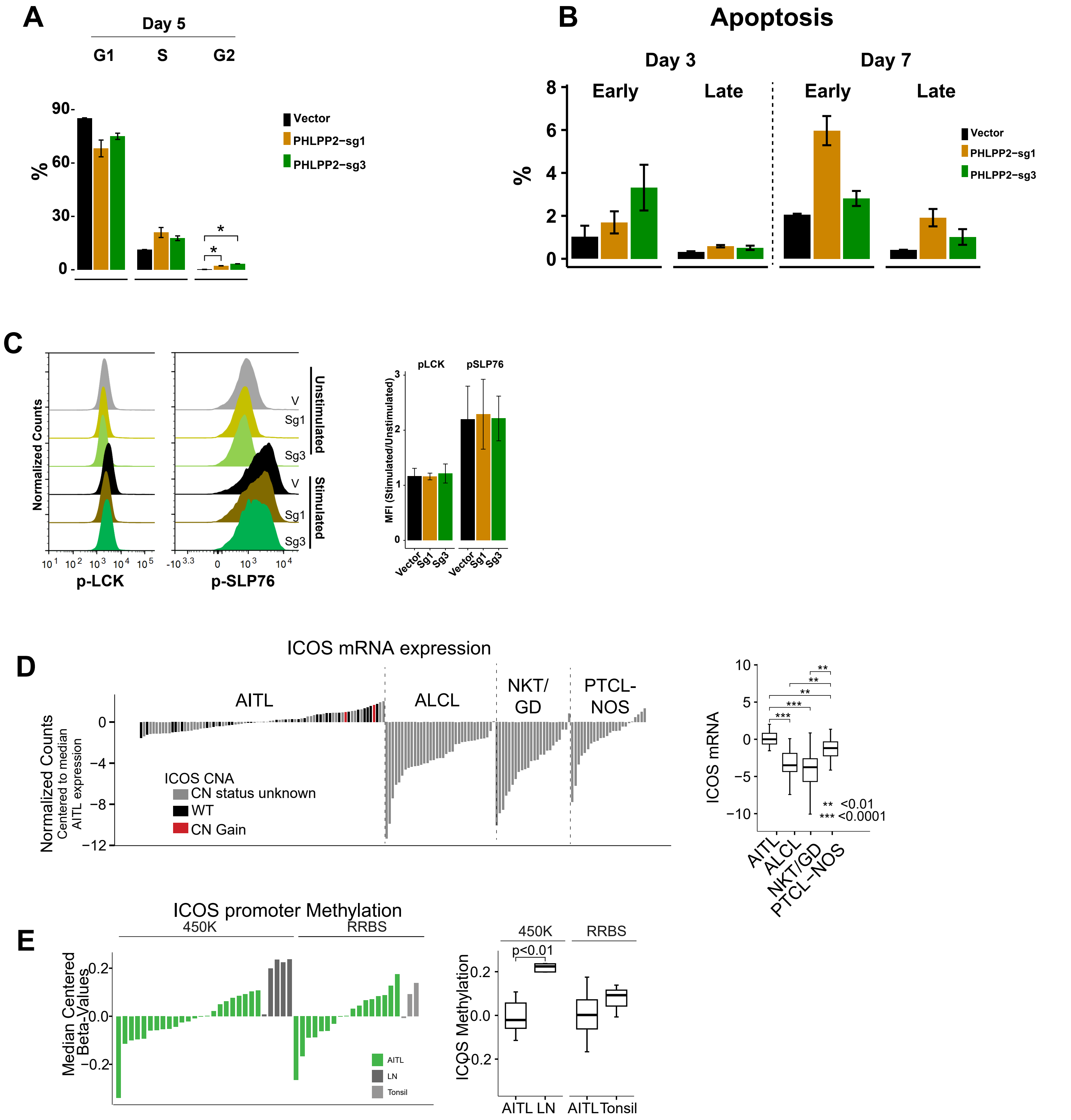


**Figure-S7.** A. Cell cycle analysis of CD4T cells with/without PHLPP2 knock-out cultured in standard culture conditions (containing IL-2 and αCD3/αCD28) at day 5 post-plating in fresh media. B. Apoptosis analysis of CD4T cells with/without PHLPP2 knock-out cultured in standard culture conditions (containing IL-2 and αCD3/αCD28) at days 3 and 7 post-plating in fresh media. C. Phosphorylation status of LCK and SLP76 measured by flow cytometry in unstimulated or cells stimulated for 10 minutes with 10ug of soluble αCD3/αCD28. The data are from a representative experiment of 2-3. The median fluorescent intensity (MFI) of stimulated/unstimulated is shown in the bar plot on the right. Asterisks denote signficant differences, * $p<0.05$. D. Association of ICOS mRNA expression with CN status in AITL. Expressions in other PTCL entities are shown for comparison. The data shows that AITLs have higher ICOS mRNA expression compared to other entities and cases with 2q33.2 CN gain have high mRNA expression. E. Comparison of the methylation levels of the ICOS promoter region in AITLs and normal control lymph nodes or tonsils with CN status in AITL.
