# Supplementary material for: Integrative Genomic and Transcriptomic Analysis Reveals Targetable Vulnerabilities in Angioimmunoblastic T‐Cell Lymphoma
Source: Am J Hematol. 2025 Jun 13;100(9):1486–501. doi: 10.1002/ajh.27736 (PMC12326222; doi:10.1002/ajh.27736)
Supplement: Supplementary file 1 — Data S1. Supporting Information. [file AJH-100-1486-s001.zip › AITL_AJH.Supplment.FINAL.033125.docx]

**Supplementary Methods**

Patient specimen

174 AITLs with WES (n=124) and/or RNAseq (n=78) and/or methylation profiling (n=40) were included in the study. Cases that were part of the I-PTCL study^1,2^ or LLMPP^3^ had central pathology review that was detailed in the previously published studies. The cases collected from previously published literature relied on the published diagnosis. The clinical characteristics such as age, gender, treatment and stage as well as type of data available are shown in **Table-S2/Figure-S1A**. IPI was only available for 27 of the cases and thus was not further analyzed. Outcome on the basis of age (>50 years vs <50 years and >60 years and <60 years) and gender, as well as mutation status of commonly mutated genes (*TET2, RHOA, IDH2, DNMT3A, CD28, PHLPP2*) was assessed. The DNA and RNA from the diagnostic biopsies were used for somatic variant detection and expression analyses, whereas blood or buccal swab DNA, if available, was used for exclusion of germline variants. While fresh-frozen DNA and corresponding RNA was used for whole exome-sequencing (WES) and RNA-seq respectively, some additional AITLs previously published (n=26) were included as shown in **Table-S2**^4,5^, although a large number of such cases had <50X coverage (see **Figure-S2A**). A total of 119 AITLs were evaluated for WES analysis, RNA-seq data were assessed for 78 cases. Of the 78 cases with RNA-seq data, 24 AITLs had microarray transcriptomic data (HG-U133plus2 , Affymetrix Inc) from earlier studies^1,6^ and were used to bridge the data from the microarray platform to the RNA-seq platform for cross-validation. This analysis also resulted in 5 PTCL-NOS cases with AITL-like molecular features including mutation spectrum concordant with AITL, particularly IDH2^R172^ and RHOA^G17V^ to be included in the analysis. RNA-seq data from other PTCL subtypes (e.g. ALCL (n=35), PTCL-NOS (n=34), NKCL/γδPTCL (n=17) or T-cell lines^7-9^, normal CD4+T or T-helper subsets were included for comparative analysis^10^. Other than these, we also propagated 12 AITL cases as patient-derived xenografts for a minimum of 1 passage (range: 1-5), and corresponding RNA-seq and WES data were examined for clonal evolution in NSG mice and correlated with data from primary biopsies. This study was approved by the Institutional Review Boards of UNMC and other contributing sites.

Flow sorting of neoplastic, B, and myeloid cell fractions from AITLs

Five live frozen AITL tumors and 5 AITL OCT tissues were flow sorted to obtain tumor (CD3^+^/PD1^+^), myeloid (CD68^+^, CD11b^+^), and B-cell fractions (CD79B^+^/CD3^-^). For OCT tissues, tissues were hand cut from the block, fixed in 1.5% formaldehyde for 15 minutes at room temperature. Crosslinking was quenched by adding glycine (0.125M) at 4 degrees. Tissues was ground with a pestle and pushed through a 40 um filter to obtain single cell suspensions and washed and resuspended in PBS containing 1% BSA. Cells were Stained with anti-CD3-PE (UCHT1, STEMCELL Technologies), anti-PD-1-FITC (EH12.2H7, Biolegend), anti-CD68-APC (Y1/82A, Biolegend), anti-CD11b-APC/Cyanine7 (M1/70, Biolegend), and anti-CD79B-PE/Cyanine7 (CB3-1, Biolegend) antibodies for 30 minutes at room temperature. Cells were washed 2 times and resuspended in PBS containing 1% BSA. Cells were sorted on a Bigfoot Spectral Cell Sorter (Invitrogen). DNA was extracted as previously described and a portion was subjected to whole genome amplification using the Repli-G mini kit (Qiagen, cat # 150023). Whole exome sequencing was performed on the un-amplified DNA from the tumor fraction for 9 of the sorted AITLs. One OCT sample did not have enough DNA for WES, but the amplified DNA was used for sanger sequencing. Sanger sequencing of identified TET2, IDH2, DNMT3A, RHOA, PLCG1, TET3, and PHLPP2 was performed to determine presence in non-tumor fraction (myeloid and/or B-cells). TCR-gamma chain rearrangement as assessed by PCR/capillary electrophoresis^11^ was performed to determine clonality of the tumor specimen.

DNA and RNA isolation and library preparation for high-throughput analysis

Total RNA and g-DNA from fresh-frozen tissues were extracted using DNeasy Blood and Tissue Kit (Qiagen Inc, MD) as per manufacturer’s guidelines. Quantity and quality of the DNA was examined using Agilent Bio-analyzer and Qubit fluorometric quantitation. The g[enomic DNA](https://www.sciencedirect.com/topics/neuroscience/genomic-dna) was sheared to 250 bp, end-repaired, A-tailed, and ligated to Illumina paired-end adapters, and amplified. The samples were column purified, quantified, and hybridized overnight to baits provided in the SureSelect Human All Exon 50 MB kit (Agilent Technologies). The captured libraries were amplified and sequenced on Illumina sequencer (HiSeq2500 or NextSeq550), and the whole exome-seq (WES) was performed as described^12^. In addition, we used RNA with a RIN value >8 for RNA-seq library preparation, and at least 1-2μg of total RNA was converted to mRNA libraries using the Illumina mRNA TruSeq kit according to the manufacturer’s directions. Libraries were sequenced 48×7×48bp on the Illumina HiSeq 2000 as previously described^13^. For samples collected from the literature, the RNA-seq capture method of each sample is described in **Table-S2**.

Whole-exome sequencing and RNA-seq analysis

We included 124 AITLs for WES and 78 AITLs for RNA-seq, with overlapping RNA-seq and WES in 31 cases, and methylome data in 40 AITL cases (see below). We included an additional published subset of AITLs, either downloaded from the genomic SRA database (NCBI), or acquired from the respective institution through a material transfer agreement^4,6^. The sample origin and sequencing coverage are denoted in **Table-S2,** which also indicates 29 low-depth cases that were included for variant discovery, but excluded for frequency calculation purposes. Adapter sequences and poor-quality WES reads were subject to trimming with Trimmomatic (v0.36). Reads were further mapped to the human genome (hg19) using BWA-MEM (v0.7.15), and duplicate reads were marked with Picard (v2.4.1). Genome Analysis Toolkit (v3.6) was used for local realignment and base quality recalibration, with variant calling and filtering performed using VarScan2 (v2.4.0). The variants were annotated using Annovar (20180416, http://annovar.openbioinformatics.org). Variants retained were supported by at least four reads in the tumor sample, with a minimal variant allele fraction (VAF) of 5%, and with variant reads present on both the plus and minus strand. Variants were further excluded if they satisfied either of the following criteria: 1) existed in the dbSNP138NotFlagged database (<http://annovar.openbioinformatics.org/en/latest/user-guide/filter>), 2) were present at >1% in gnomAD non-cancer sample database (<https://gnomad.broadinstitute.org/>), 3) were in a region of segmental duplication (SuperDups, Annovar), 4) did not change protein-coding sequence or affect canonical splice sites, 5) were recurrent in an unrelated set of 91 normal samples^14^, 6) were shared by over 11 cases in unfiltered data, unless it was a known mutation (reported in COSMIC). Finally, hotspot mutations affecting IDH2^R172^, RHOA^G17^ , and CD28^T195, D124, or F51^ with VAF <5% were rescued which accounted for (7 of 28,10 of 63 , and 4 of 14 mutations respectively). After application filters designed to remove polymorphisms, unpaired cases still had ~2x more variants than paired cases, suggesting contamination by private germline SNPs. Thus, variants the VAF greater than 2x the standard deviation from the mean VAF for that case, were annotated as “possible germline”.

The RNA-seq analysis was performed on 78 AITL samples, which included 20 samples obtained from the SRA database, while 10 samples from normal T cells (naïve, effector, and T_FH_) were also used^10^. Several other PTCL entities, normal B cells, and follicular lymphomas described were included for comparative analysis^4^. Quality of the raw reads was assessed by FASTQC (v 0.11.7), and sequences from contaminating adapters removed with Trimmomatic (v0.36). Trimmed reads were then aligned to the hg19 genome using STAR (v 2.5.3)^15^ and gene expression quantification was done using HTSeq (v.0.9.1)^16^. Gene expression normalization was done with the R-package DESeq2 (1.24.0) or by the FPKM method. T-cell receptor and immunoglobulin clonality analysis was performed with MiXCR (v.3.0.13), as previously described^4^. Fusioncatcher (<https://github.com/ndaniel/fusioncatcher>)^17^ was used for fusion analysis and viral genome expression detection. Published data is available in the SRA database or can be obtained by contacting the authors. Mutations affecting genes that were expressed in 10 normal T-cell samples (FPKM>2) were analyzed by pathway-enrichment tools (DAVID, v6.7).

Mutation status of the RNA-seq cases were assessed for genes found to be commonly mutated in the WES samples. The STAR-aligned BAM file was further processed for variant calling, by marking duplicate reads with Picard (v2.4.1) and subsequently AddOrReplaceReadGroups, SplitNCigarReads, local realignment and base quality recalibration was performed using Genome Analysis Toolkit (v3.6).

Variant calling was performed with VarScan2 (v2.4.0) and the variants were annotated using Annovar (20180416, <http://annovar.openbioinformatics.org>). Mutation filtering was performed as described for the WES above and additionally any variant found in the RNA-seq data of the 10 normal T-cells were excluded and only mutations in genes already known to be mutated in the WES were assessed. Mutations of CD28 identified by RNA-seq were used in Figures 2C and S5D.

Gene Expression Profiling Analysis

Gene expression profiles of AITLs or other PTCLs were analyzed for molecular diagnosis using previously published signatures ^1,6^. Other computational tools like CIBERSORT^18^ and xCell^19^ were used to infer cell type composition. GSEA (<https://www.gsea-msigdb.org/gsea/index.jsp>) using GSEA databases and the lymphoid signature database (https://lymphochip.nih.gov/signaturedb/) for gene signature or pathway analysis. For differential gene expression analysis of AITL compared to other PTCL a subgroup of 39 AITLs and 74 PTCLs that prepared with a polyA RNA-seq protocol to avoid any differences based on protocol.

Immunhistochemisty scoring of B and Myeloid Markers

FFPE tissue microarrays from cases included in previous studies^3,20^ were evaluated for B-cell (CD20), macrophage (CD68 and CD163) by immunohistochemistry, slides were scanned at 40x magnification, and the lymphoid areas were annotated (soft tissue was excluded). The annotated slides were analyzed using QuPath 0.3^21^ with StarDist^22^ with custom trained models for cytoplasmic and nuclear immunostains as well as in situ hybridization stains to segment the cells. The slide percent positivity was determined by overlaying a grid in the annotated area and excluding squares in the grid with low cell density, which often represent vessels or intermixed soft tissue. The remaining squares were averaged to arrive at the total for the slide or tissue microarray position (**Table-S9**).

Genome-wide methylation profiling

DNA (200-500ng) was bisulfite-converted using EZ DNA methylation kit (Zymo Research) and genome-wide methylation profiling was performed using Infinium HumanMethylation450 BeadChip array (Illumina, San Diego, CA) according to the manufacturer's protocol. The array was stained fluorescently, and scanned with an iSan System (Illumina), and the data were analyzed by GenomeStudio Methylation Module Software (Illumina). Raw intensity data files (idat files) were processed using the R package Rnbeads (https://rnbeads.org/). A CpG site was informative if the sum of the signals for methylated and unmethylated sequence at the CpG site was significantly higher (detection p-value < 0.05) than signals of the negative control probes on the same array. For each CpG site, the β-value reflects the methylation level, which was computed by β = (max (M, 0))/(|U| + |M|+100). A β-value of 0–1.0 indicates the percent methylation from 0% to 100%, respectively. We also analyzed the data of 17 additional AITL cases, 3 Tonsils, and 4 CD4 T cell samples with reduced representation bisulfite sequencing for methylation analysis from our earlier series for validation[^22^](#_ENREF_22) using the Rnbeads packages. To identify differentially methylated candidate genes, genes whose promoters were consistently differentially methylated between AITLs and normal controls ( Lymph nodes for the Infinium HumanMethylation450 BeadChip array and tonsils for the RRBS data), where at least one of the datasets had an FDR p-values of <-0.05, were identified. Pathways consistently hypo- or hyper-methylated were assessed by concensusPathDB (http://cpdb.molgen.mpg.de/). To more stringently filter the differentially methylated genes the methylation beta-values was correlated with the mRNA expression values for the AITL cases. The HumanMethylation450 BeadChip array had RNA-seq profiling available, while the RRBS cases had previously published Affymetrix U133plus2 array profiling.[^22^](#_ENREF_22) As a final filter, hypomethylated genes that were upregulated in the AITLs compared to other PTCLs or hypermethylated and downregulated in AITLs compared to other PTCLs (**Figure-2**) were selected as the most biologically relevant gene set.

Genomic characterization of AITL patient-derived xenografts

Six- to eight-week-old male NSG (Nod SCID Gamma) mice were subcutaneously implanted with fresh human fetal bones of 17–19 gestational weeks (Alameda, CA, USA) (SCID/NSG-hu), with experimental procedures approved by the Institutional Animal Care at WCMC and UNMC. Approximately four to six weeks following implantation, 5 × 10^6^ freshly isolated lymphoma cells were directly injected into human fetal bone implants within SCID/NSG-hu hosts, and the growth of implants were assessed periodically either by palpation (s.c) or by magnetic resonance imaging (MRI). Mice were sacrificed at early signs of distress. Tumor engraftment was determined by monitoring circulating peripheral blood lymphocytes (by multicolor flow cytometry), and by defining size changes of multiple parenchymal organs (spleen, liver, kidney by MRI). Innate biological properties of xenografts, such as tumor growth, cell proliferation, apoptosis and dissemination potential were analyzed in vivo using luciferase transduce PDX cells and MRI and in vitro by conventional proliferation, apoptosis, and invasion assays on explanted tumors (caspase-3, Ki-67 etc).

A panel of markers characteristically expressed by AITL cells (CD10, PD-1, CXCL13 and other pan T-cell markers), human cytokine profile (human IL6, IL17), and EBV+ cells by in-situ hybridization (EBER), were evaluated for every engrafted tissue. Once tumor growth was established in the first generation (T1), the mice were sacrificed, and tumor masses were isolated. The tumor cells were tested for AITL tumor markers and then inoculated in NSG mice as the second generation (T2) and serially implanted for subsequent generations (T2-T5) of mice. Clonal evolution was determined using genetic analysis in primary and subsequent passages.

For WES and RNA-seq, library preparation was done as described above. The SureSelect V6.0 kit (Agilent Technologies) kit and TruSeq® Stranded Total RNA Library Prep Human/Mouse/Rat (Illumina) were used, respectively. Sequencing Analysis was done as described about except samples were aligned to a merged mouse (mm10) and human (hg19) reference and reads aligning to the human chromosomes were used.

The mutations from donor patients and PDX-passages, with variant allelic fraction and CNV information for each variant, were passed to PyClone-VI , bayesian statistical model , to the cellular prevalence of genomic variants and infer subclonal clusters^23^. PyClone considers variant allele frequencies in tumor samples, incorporating prior information, sequencing noise, and tumor heterogeneity to infer the cellular prevalence of each variant. Hierarchical clustering groups variants with similar prevalence patterns, providing a basis for clustering genomic variants based on their cellular prevalence. The clustering output data from Pyclone-VI was used to build a patient-PDX model that captures evolutionary trajectories from patient-PDX passages using the REVOLVER pipeline, implementing the statistical model outlined in Caravagna et al ^24^. The model is represented as a tree where nodes show cluster of mutations annotated in patient-PDX passage data, some flagged as driver gene mutations. Each cluster group can have zero, or one or more driver genes associated with it. We focused on driver gene mutations that are recurrent in multiple patient PDX passages and appear in several patients (repeated evolution), and highlighted the clusters that included driver gene mutations.

*Gene expression signature analysis:* Gene expression signature differences between passages were evaluated using DESeq2 to identify genes that were differentially expressed (p<0.05, and 2 fold expression difference) between late vs early passages (T3 vs T1 and T5 vs T1). 1604 differentially expressed genes were identified between T3 and T1, while 1523 differentially expressed genes were identified between T5 and T1. To determine if the signatures had biological significance we evaluated expression of the identified signatures in our AITL cohort. The AITL cases were divided into the upper half and lower half based upon their magnitude of expression difference of the upregulated signature genes vs the down regulated signature genes and overall survival was assessed in cases with available data. A subset of the T3 vs T1 (n=266) or T5 vs T1 (n=507) signature genes that represent genes differentially expressed between TFH and naïve T-cells were also evaluated for outcome significance in AITL cases.

Clinical outcome correlation and statistical analyses

The Kaplan-Meier method was used to estimate the overall survival distributions using the R survminer package. Overall survival times were calculated as the time from diagnosis to the date of death or last contact. Patients who were alive at last contact were treated as censored for the overall survival analysis. The log-rank test was used to compare survival distributions. All statistical tests are two-sided and p-values less than 0.05 were considered to be statistically significant unless specified otherwise.

Statistical tests and p-value cutoffs for overall survival and bioinformatics analyses are as described above. For all else, comparisons between two groups were conducted using a two-tailed Student’s t-test. P-values < 0.05 were considered significant. The data analysis for this manuscript was conducted using R (https://www.r-project.org/). Plots were generated using the R packages ggplot2, maftools, and survival.

In-vitro functional analysis of *PHLPP2* knock-out using human CD3+ or CD4+ T cell culture:

Normal CD4+ T cells were isolated from peripheral blood mononuclear cells (PBMCs) of healthy donors (Elutriation core, UNMC) or tonsils using the negative selection CD4+ T cell Isolation Kit (EasySep; Stem cell Technologies, Inc). T cell purity was analyzed by flow cytometry on an ACEA NovoCyte^TM^ flow cytometer. CD4+ T cells were maintained in culture at 0.5-1x10^6^ cells/mL in RPMI 1640 containing glutamine (300 ug/ml), 10% FBS), penicillin G (100 IU/ml), streptomycin (100μg/ml), and recombinant human IL-2 (Cell signaling Technology; # 78145; 20 ng/mL) and α-CD3/α -CD28 immunocult (Cell signaling Technology; 10971). 293T cells were maintained in DMEM supplemented with 10% (FBS), penicillin G (100 IU/ml), streptomycin (100μg/ml). Cell lines underwent routine mycoplasma testing using the Universal Mycoplasma Detection Kit (ATCC).

Vectors and stable transfections/transductions: To investigate the loss of function of PHLPP2 in T cells, previously reported CrispR-Cas9 vectors were used^4^. The lentivirus was grown in 293T cells transduced using the calcium phosphate method with the Measles virus glycoprotein-pseudotyped lentiviral packaging system as described^25,26^. Virus was collected at 48 and 72 hours, filtered through .45-micron filters, and precipitated using 10% PEG-8000. Primary T-cells were transduced with virus in the RPMI media supplemented with polybrene (10 ug/ml, Chemicon-Millipore) and spinoculated (350xg, 1.5 hour). T cells were selected in 1-2 µ/ml puromyocin for 3 days.

For apoptosis, cell-cycle, and cell-viability assays cells were plated day 0 in complete media containing IL-2 (20ng/ul) and α-CD3/α-CD28 immunocult (25 ul/mL).

Apoptosis assay: Apoptosis was quantified using a FACS-Calibur flow cytometer after staining the cells with Annexin V-PE using the apoptosis detection kit (Pharmingen, Inc) according to the manufacturer’s instructions on day 3 and 7.

Cell Cycle. On day 5 post plating in media, cells were fixed in 70% ethanol and the cell cycle profile was analyzed using propidium-iodide. The cells were washed once with PBS and re-suspended in buffer containing 1× PBS, RNAseA (10μg/ml) and PI (50ug/ml). The cells were incubated at 37°C for 15 minutes and then analyzed with ACEA NovoCyte flow cytometer.

Cell viability assay: Cell viability was performed in the 384 well plates by using PrestoBlue™ Cell Viability Reagent (In-vitrogen, Inc) per the manufacture’s protocol and read on a Tecan Infinite M200Pro.

Western blotting: The cells were lysed in lysis buffer (50 mM HEPES, pH 7.4, 150 mM NaCl, 1.5 mM MgCl_2_,1% Triton X-100, Halt Protease and Phosphatase Inhibitor Cocktail. Forty to 80μg of whole-cell extracts were separated by SDS-polyacrylamide gel electrophoresis and transferred to polyvinylidene difluoride membranes. Membranes were blocked in Tris-buffered saline with 0.1% Tween and 5% milk at room temperature for 30 minutes to 1 hour and then incubated with primary antibody (**Table-S10**) at 4°C overnight, followed by treatment with secondary antibodies. The immunoblots were visualized using a Bio-Rad, Chemi Doc^TM^ MP. Antibodies used for Western blotting and Flow cytometry are listing in **Table-S10**.

Flow cytometry:0.0.25x10^6^ cells were washed twice in PBS and suspended in 100μl staining buffer (1x PBS, 0.5% BSA, and 2 mM EDTA), containing antibodies for surface marker staining, CD4, CXCR5, and PD1 for 1 hour at 4°C. Cells were then washed twice in staining buffer and run on a NovoCyte 2060R with data analysis using the NoVo Express software. For TCR activation analysis, CD4 T cells were cultured in media without IL-2, α-CD3, and α -CD28 for 24 hours. Cells (0.25x10^6^) were left unstimulated or stimulated with 10 ug α -CD3 and 10 ug α -CD28 for 10 minutes and then immediately fixed in prewarmed BD Phos-flow fix buffer (#557870) and permeabilized with BD Phosflow Perm buffer III (#558050) according to the manufacturer's instructions. Cells were then stained overnight with antibodies to Lck (pY505) (BD #557879), SLP76 (pY128) (BD #558437), and Zap70 (pY319)/Syk (pY352) (BD #557817), washed with staining buffer 2x and then ran on a NovoCyte 2060R.

In-vitro functional analysis of *TET2 knock-out* using human CD4+ T cells:

Peripheral blood mononuclear cells were isolated from healthy donors by density gradient centrifugation. CD4 T cells were then purified by using the CD4 T Cell Isolation kit (STEMCELL). T cells were cultured in X-Vivo 15, 10% FBS, 5 ng/ml IL-7 and 200 U/ml IL-2 and stimulated 2 days with CD3/CD28 T cell activator Dynabeads (Invitrogen) at 1:1 ratio before RNP electroporation. RNPs were formed by the addition of SpCas9 nuclease (IDT) with 3 single gRNA targeting TET2 (Synthego) at 37℃ for 15 minutes (sequences below). Cells were expanded in X-Vivo 15, 10% FBS, 50 U/ml IL-2 with CD3/CD28 T cell activator Dynabeads (Invitrogen) at 1:0.5 ratio. The Dynabeads were refreshed at 10 days interval, representing one cycle.

PI3K/AKT/mTOR signaling activation with α-ICOS stimulation

For examination of the PI3K/AKT/mTOR signaling pathway, previously activated CD4 T cells (four times) were beads removed and kept resting for 5 days, which were then treated with 1 ug/ml α-CD3 (Biolegend, 300401) and 4 ug/ml α-ICOS (Biolegend, 313502) for 2 days. Antibodies including Phospho-PI3 Kinase p85 (Tyr458)/p55 (Tyr199) (CST, 4228); Phospho-Akt (Ser473) (CST, 4060S); AKT (CST, 9272); Phospho-S6 Ribosomal Protein (Ser235/236) (CST, 4858S), TET2 (abcam, ab124297) were used for western blot.

Flow cytometry

ICOS expression was measured with Brilliant Violet 421™ anti-human/mouse/rat CD278 (ICOS) Antibody (Biolegend, 313524) according to the manufacturer’s protocol. Data were analyzed by FlowJo v10.1 (BD).

sgRNA Sequences:

5’-AGAGCUCAUCCAGAAGUAAA

5’-UUAUGGAAUACCCUGUAUGA

5’-UCCUCCAUUUUGCAAACACU

Supplemental Results

Somatic mutations identification in AITL.

Genes affected by mutation only in the unpaired cases are noted (**Table-S3).** All mutations that passed the filtering criteria are shown in **Figure-S2**. Despite the application of multiple variant filters and SNP databases, unpaired cases had a higher average mutation burden than paired cases (56 versus 26 per case, respectively), indicating expected contamination by low-frequency or private germline variants. Possible germline variants based on their VAF distribution are annotated (**Table-S3**). Overall, we detected 5,105 variants in 119 cases affecting 3,636 genes (**Table-S3**). SNVs (n=4,409) were the major mutation type at >6-fold higher occurrence than insertions/deletion (n=696), suggesting that SNVs are the major mutational drivers of AITL lymphomagenesis. SNVs included missense mutations (4,093/4,409 or 92.8%), nonsense mutations (217/4,409 or 4.9%), and splice site variants (99/4,409 or 2.2%), and primarily affected G/C > A/T alteration (**Figure-S2C-G**). Indels included frameshifts (427/696, 61.3%), non-frameshifts (243/696, 34.9%), splice site variants (12/696, 1.7%), and nonsense mutations (14/696, 2%)].

Integrative analysis of transcriptomic and mutation data

Our integrative analysis revealed that 37% of the WES variants could also be identified in corresponding RNA-seq (**Table-S4**). Mutations leading to rapid mRNA degradation would not be captured by RNA-seq, and mutations in low-expressed genes may not be detectable. However, we validated >90% of hotspot mutations (e.g., *IDH2^R172^* and *RHOA^G17V^*) identified from WES in the RNA-seq data, in addition to other frequent mutations in *TET2* (79%) and DNMT3A (100%) (**Table-S4**), suggesting that SNV detection were robust in both WES and RNA-seq data. Mutation analysis identified epigenetic dysregulation, TCR, and PI3K signaling as major aberrant genetic pathways, and corresponding RNA-seq analysis revealed enrichment of similar oncogenic signaling pathways (e.g., TCR, PI3K, and NF-κB).

Of the mutants detected from WES, *DNMT3A* and *TET2* showed skewed VAF distribution compared to other commonly mutated genes (*RHOA^G17V^*, *IDH2^R172^*, *PLC*G1, or *CD28*), lending support to the idea that *TET2* and *DNMT3A* mutations often originate in hematopoietic stem cells (HSC). Similar skewed correlation was observed when compared to TCR-α/β clonal estimations. We performed in-silico tumor milieu analysis using CIBERSORT in order to assess whether a distinct cellular fraction is enriched in cases with higher *TET2* mutation VAF. Lower naïve B cell content in the *TET2* VAF-high group compared to the *TET2* wild-type (**Table-S11**) was observed, and was validated in another cohort with HG-U133+2 transcriptomic data and DNA-amplicon sequencing *TET2* data^27,28^. Memory B-cell content was lower in AITLs with high-VAF *DNMT3A* mutations as compared to wild-type cases (**Table-S11**), suggesting functional impact on B cell differentiation in lymph nodes, if *TET2* and *DNMT3A* deficiency occurs in progenitor cells.

References

1. Iqbal J, Weisenburger DD, Greiner TC, et al. Molecular signatures to improve diagnosis in peripheral T-cell lymphoma and prognostication in angioimmunoblastic T-cell lymphoma. *Blood*. 2010;115(5):1026-1036.

2. Vose J. International peripheral T-cell and natural killer/t-cell lymphoma study: pathology findings and clinical outcomes. *J Clin Oncol*. 2008;26(25):4124-4130.

3. Iqbal J, Wright G, Wang C, et al. Gene expression signatures delineate biological and prognostic subgroups in peripheral T-cell lymphoma. *Blood*. 2014;123(19):2915-2923.

4. Gong Q, Wang C, Zhang W, et al. Assessment of T-cell receptor repertoire and clonal expansion in peripheral T-cell lymphoma using RNA-seq data. *Sci Rep*. 2017;7(1):11301.

5. Rohr J, Guo S, Huo J, et al. Recurrent activating mutations of CD28 in peripheral T-cell lymphomas. *Leukemia*. 2016;30(5):1062-1070.

6. Abate F, Todaro M, van der Krogt JA, et al. A novel patient-derived tumorgraft model with TRAF1-ALK anaplastic large-cell lymphoma translocation. *Leukemia*. 2015;29(6):1390-1401.

7. Kucuk C, Jiang B, Hu X, et al. Activating mutations of STAT5B and STAT3 in lymphomas derived from gammadelta-T or NK cells. *Nat Commun*. 2015;6:6025.

8. Yoo HY, Sung MK, Lee SH, et al. A recurrent inactivating mutation in RHOA GTPase in angioimmunoblastic T cell lymphoma. *Nat Genet*. 2014;46(4):371-375.

9. Palomero T, Couronne L, Khiabanian H, et al. Recurrent mutations in epigenetic regulators, RHOA and FYN kinase in peripheral T cell lymphomas. *Nat Genet*. 2014;46(2):166-170.

10. Weinstein JS, Lezon-Geyda K, Maksimova Y, et al. Global transcriptome analysis and enhancer landscape of human primary T follicular helper and T effector lymphocytes. *Blood*. 2014;124(25):3719-3729.

11. Greiner TC, Rubocki RJ. Effectiveness of capillary electrophoresis using fluorescent-labeled primers in detecting T-cell receptor gamma gene rearrangements. *J Mol Diagn*. 2002;4(3):137-143.

12. Moffitt AB, Ondrejka SL, McKinney M, et al. Enteropathy-associated T cell lymphoma subtypes are characterized by loss of function of SETD2. *J Exp Med*. 2017;214(5):1371-1386.

13. Gong Q, Wang C, Rohr J, Feldman AL, Chan WC, McKeithan TW. Comment on: Frequent CTLA4-CD28 gene fusion in diverse types of T-cell lymphoma, by Yoo et al. *Haematologica*. 2016;101(6):e269-270.

14. Bouska A, Bi C, Lone W, et al. Adult high-grade B-cell lymphoma with Burkitt lymphoma signature: genomic features and potential therapeutic targets. *Blood*. 2017;130(16):1819-1831.

15. Dobin A, Davis CA, Schlesinger F, et al. STAR: ultrafast universal RNA-seq aligner. *Bioinformatics*. 2013;29(1):15-21.

16. Anders S, Pyl PT, Huber W. HTSeq--a Python framework to work with high-throughput sequencing data. *Bioinformatics*. 2015;31(2):166-169.

17. Nicorici D, Şatalan M, Edgren H, et al. <strong>FusionCatcher</strong> – a tool for finding somatic fusion genes in paired-end RNA-sequencing data. *bioRxiv*. 2014.

18. Newman AM, Liu CL, Green MR, et al. Robust enumeration of cell subsets from tissue expression profiles. *Nat Methods*. 2015;12(5):453-457.

19. Aran D, Hu Z, Butte AJ. xCell: digitally portraying the tissue cellular heterogeneity landscape. *Genome Biol*. 2017;18(1):220.

20. Cannatella J, Sharma, S., Bouska, A., Greiner, T., Damore, F.,, Pedersen M, Ong, C., Rosenwald, A., Ott, G., Vose, J., Weisenburger, D.,, Chan WC, Iqbal, J., Amador, C. B-cell Enriched Tumor Microenvironment Predicts Favorable Prognosis in

Angioimmunoblastic T-cell Lymphoma (AITL). *USCAP Abstracts 2021*. 2021:673.

21. Bankhead P, Loughrey MB, Fernandez JA, et al. QuPath: Open source software for digital pathology image analysis. *Sci Rep*. 2017;7(1):16878.

22. Schmidt U, Weigert M, Broaddus C, Myers G. Cell Detection with Star-Convex Polygons. Medical Image Computing and Computer Assisted Intervention – MICCAI 2018. Cham: Springer International Publishing; 2018:265-273.

23. Gillis S, Roth A. PyClone-VI: scalable inference of clonal population structures using whole genome data. *BMC Bioinformatics*. 2020;21(1):571.

24. Caravagna G, Giarratano Y, Ramazzotti D, et al. Detecting repeated cancer evolution from multi-region tumor sequencing data. *Nat Methods*. 2018;15(9):707-714.

25. Frecha C, Lévy C, Costa C, et al. Measles virus glycoprotein-pseudotyped lentiviral vector-mediated gene transfer into quiescent lymphocytes requires binding to both SLAM and CD46 entry receptors. *J Virol*. 2011;85(12):5975-5985.

26. Zhou Q, Schneider IC, Gallet M, Kneissl S, Buchholz CJ. Resting lymphocyte transduction with measles virus glycoprotein pseudotyped lentiviral vectors relies on CD46 and SLAM. *Virology*. 2011;413(2):149-152.

27. Wang C, McKeithan TW, Gong Q, et al. IDH2R172 mutations define a unique subgroup of patients with angioimmunoblastic T-cell lymphoma. *Blood*. 2015;126(15):1741-1752.

28. Wang M, Zhang S, Chuang SS, et al. Angioimmunoblastic T cell lymphoma: novel molecular insights by mutation profiling. *Oncotarget*. 2017;8(11):17763-17770.
